# High-temperature and high-salinity performance of chelating agents for oilfield scale inhibition with dynamic scale loop

**DOI:** 10.1038/s41598-026-49582-0

**Published:** 2026-04-28

**Authors:** Ose Budiman, Shabeeb Alajmei, Murtada Saleh Aljawad, Mohamed Mahmoud, Muhammad Shahzad Kamal, Mobeen Murtaza, Prasad Karadkar

**Affiliations:** 1 Department of Petroleum Engineering, College of Petroleum Engineering and Geosciences, King Fahd University of Petroleum and Minerals (KFUPM), 31261 Dhahran, Eastern Province Saudi Arabia; 2 Center for Integrative Petroleum Research, King Fahd University of Petroleum and Minerals, 31261 Dhahran, Eastern Province Saudi Arabia; 3 Production Technology Division, EXPEC Advanced Research Center, Saudi Aramco, 31311 Dhahran, Saudi Arabia

**Keywords:** Oilfield scale, Chelating agent, Dynamic scale loop, High-temperature and high-salinity, Seawater, Chemistry, Environmental sciences

## Abstract

Scale formation in oil and gas operations reduces production efficiency and increases costs, especially in high-salinity and high-temperature environments. Conventional inhibitors often fail in such harsh conditions. This study examines diethylenetriaminepentaacetic acid (DTPA) and glutamic acid diacetic acid (GLDA) as primary scale inhibitors under dynamic flow conditions. The experiments use a dynamic scale loop (DSL) that mimics reservoir conditions, with a commercial phosphonate scale inhibitor acting as a comparison benchmark. Experiments were performed at temperatures of 200 °F, 275 °F, and 338 °F, using high salinity water (TDS of 58,500 ppm) representing seawater and formation water (TDS of 274,740 ppm) at mixing ratios of 50:50 and 80:20, with injection rates of 1 and 10 cc/min, and inhibitor concentrations ranging from 0.1 to 1.0 wt%. Scale formation was strongly accelerated by increasing temperature, flow rate, and ion interaction in the 50:50 brine mixture. All inhibitors delayed scale formation relative to the uninhibited case; however, performance varied significantly with operating conditions. Inhibitor effectiveness was evaluated using a normalized improvement factor relative to prescale conditions. DTPA demonstrated strong performance at elevated temperatures, while GLDA showed more consistent performance at higher flow rates. The phosphonate inhibitor performed well under moderate conditions but showed reduced effectiveness under more severe HTHS conditions. Notably, GLDA at 338 °F, with a 50:50 brine mixture and a flow rate of 10 cc/min, delayed scale formation by 11-fold, the most significant improvement observed. These findings demonstrate that inhibitor performance is governed by both thermodynamic stability and kinetic effects and highlight the importance of condition-specific inhibitor selection. Overall, this study provides a systematic framework for evaluating scale inhibitors under realistic dynamic HTHS conditions and demonstrates the potential of chelating agents as effective alternatives for challenging environments.

## Introduction

Scale formation in oil and gas reservoirs is a common operational issue that hinders production, reduces formation permeability, and increases maintenance costs. It occurs due to the precipitation of insoluble salts, such as calcium carbonate, barium sulfate, and strontium sulfate. Caused by changes in temperature, pressure, and fluid mixing. These issues are even more severe under high-temperature and high-salinity (HTHS) conditions, where elevated ion concentrations and thermal gradients boost nucleation and crystal growth, making traditional mitigation methods less effective^1–3^.

Conventional scale inhibitors, predominantly phosphonate and polymer-based compounds, are widely used to mitigate these deposits. However, their application in high salinity condition is often limited by poor thermal stability, incompatibility with high concentrations of divalent cations, and precipitation tendencies at elevated temperatures^4–7^. These limitations have prompted a growing interest in alternative scale inhibitors that can withstand extreme reservoir conditions.

Recent attention has turned to chelating agents, particularly diethylenetriaminepentaacetic acid (DTPA) and glutamic acid diacetic acid (GLDA), due to their firm and easy bonding with scale-forming ions (e.g., Ca^2+^, Ba^2+^, Sr^2+^), broad pH stability (~ 3 to ~ 12), and thermal resistance up to 200 °F^8–11^. While traditionally used for scale removal, their potential as primary scale inhibitors, especially under dynamic, high-temperature, high-salinity flow conditions representative of reservoir conditions, remains underexplored. Laboratory-scale methods such as static bottle tests, tube-blocking tests, and coreflood experiments have long been used to evaluate inhibitor performance under controlled conditions^12–14^.

In a recent study, Budiman et al.^15^ provided the first systematic evaluation of DTPA and GLDA under static high-temperature conditions, reporting promising inhibition efficiencies across different concentrations. Building on that work, the current study aims to investigate the performance of these chelating agents under dynamic flow conditions, where the influence of temperature, salinity, mixing ratio, and flow rate better represents downhole scaling scenarios. The static bottle test, while simple and cost-effective for initial screening, lacks real-time quantification and does not reflect the hydrodynamic complexities of field-scale operations. Complementary analytical techniques such as X-ray diffraction (XRD), scanning electron microscopy (SEM), and inductively coupled plasma (ICP) analysis can characterize scale composition and ion concentrations, but they require offline sampling, are costly, and are not suitable for continuous monitoring^16,17^.

This study employs a dynamic scale loop (DSL), a laboratory apparatus designed to mimic reservoir conditions by continuously circulating fluid through a heated and pressurized system^18,19^. The closed-loop design enables real-time monitoring of pressure differentials, with an increase in pressure indicating the formation of scale. This setup allows for precise control over key parameters, including fluid composition, flow rate, and temperature, providing a comprehensive platform for evaluating inhibitor performance.

This study presents a systematic evaluation of DTPA and GLDA as primary scale inhibitors under HTHS conditions. This work integrates the combined effects of temperature, flow rate, brine mixing ratio, and inhibitor concentration using a DSL. The results provide new insights into flow-dependent inhibition behaviour and the comparative performance of chelating agents and phosphonate inhibitors under harsh reservoir conditions.

## Methods

This section describes the experimental design used to evaluate the performance of scale inhibitors under high-temperature, high-salinity flow conditions.

### Materials

Synthetic brines were prepared to represent high-salinity seawater (SW) as an injection water and formation water (FW), with total dissolved solids (TDS) of 58,500 ppm and 274,740 ppm, respectively. SW obtained from the Arabian Gulf has a pH of 7.2, while FW from a Saudi formation has a pH of 6.1 under standard conditions. The ionic compositions are provided in Table 1.

**Table 1:** Ion composition of the synthesized brine.

Ions	Seawater (ppm)	Formation water (ppm)
Sodium	17,490	78,100
Chloride	32,710	160,000
Calcium	600	249,000
Magnesium	2120	1830
Bicarbonate	150	< 100
Sulphate	4700	190
Potassium	780	3890
Barium	< 100	3830
Strontium	< 100	2000
Carbonate	< 100	< 100
TDS	58.500	274,740

Three scale inhibitors were evaluated, including two aminopolycarboxylate chelating agents, DTPA and GLDA, along with a commercial phosphonate-based scale inhibitor. The strength of metal–ligand interactions can be quantified using the 1:1 formation (stability) constant, expressed as:

1 $${\mathrm{M}}^{2 + } + {\mathrm{L}}^{{{\mathrm{n}} - }} \rightleftharpoons {\mathrm{ML}}^{{\left( {2{\mathrm{n}}} \right)}}$$

2 $${K}_{f}=\frac{\left[ML\right]}{\left[M][L\right]}$$

where *M*^2+^ represents the metal ion (Ca^2+^, Sr^2+^, or Ba^2+^) and *L*^*n*−^ is the fully deprotonated ligand (DTPA or GLDA). The reported values correspond to intrinsic stability constants (log_10_Kf ≡ log_10_KML), typically determined at 25 °C and ionic strength (I) ≈ 0.1 M. At 25 °C and I ≈ 0.1 M, DTPA binds Ca^2+^/Sr^2+^/Ba^2+^ far more strongly (log_10_Kf ≈ 10.7/9.7/8.8) than GLDA (log_10_Kf ≈ 5.9/4.0/3.6), corresponding to roughly 10^4^–10⁶ × larger Kf depending on the cation^20^.

The chelating agents were supplied in alkaline form (pH 12–13), whereas the phosphonate inhibitor was supplied near neutral pH (~ 7). All inhibitors were applied at concentrations of 0.1–1.0 wt%. Although the chelating agents were in alkaline form, the initial mixed solutions exhibited near-neutral pH values (6–7), as confirmed by measurement. This is due to the high ionic strength and buffering capacity of the brines, which dominate the solution chemistry at the low inhibitor dosages used.

### Experimental conditions

**Fig. 1:**
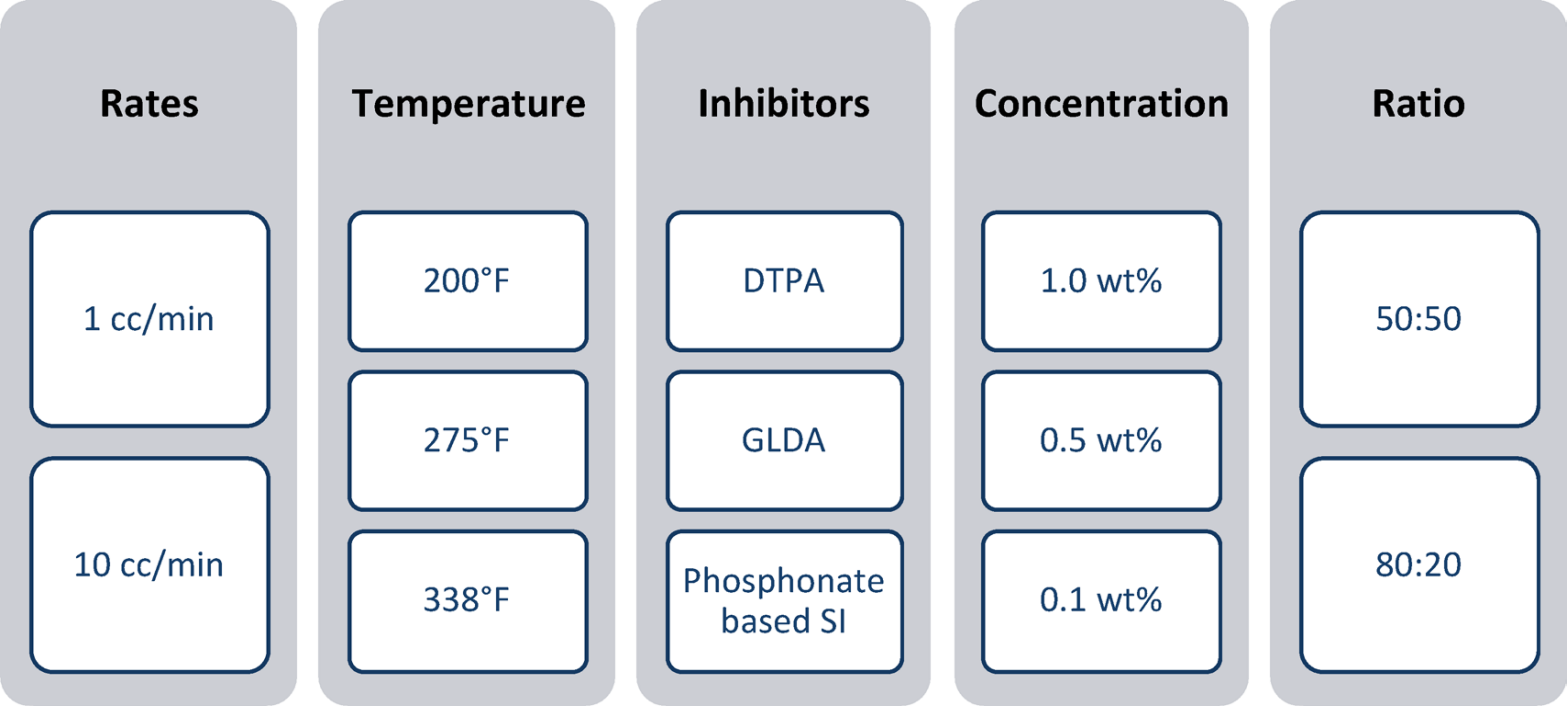
Experiment matrix.

A test matrix was designed to assess the effects of temperature, brine composition, flow rate, and inhibitor concentration on scale formation dynamics, as shown in Fig. 1.

Three temperatures (200 °F, 275 °F, and 338 °F) were selected to represent typical to elevated reservoir conditions, where scaling tendency increases with temperature. Two brine mixing ratios were evaluated: a 50:50 mixture, representing a worst-case scenario with maximum ion interaction and supersaturation potential, and an 80:20 mixture, which reflects a more typical field case with seawater-dominant conditions.

**Fig. 2:**
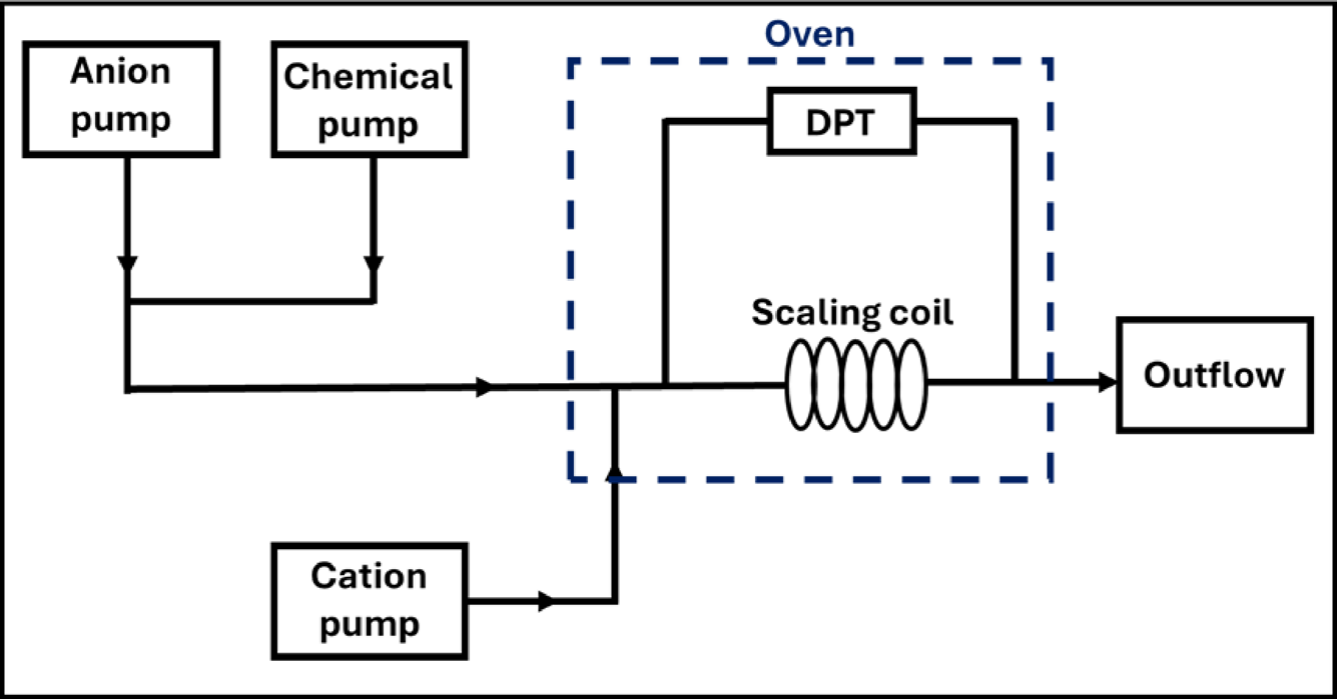
Dynamic scale loop schematic.

Total flow rates were set at 1 cc/min and 10 cc/min. The lower rate was intended to allow sufficient residence time, while the higher rate represents fast-flowing injection and flowback scenarios, where scaling is more influenced by kinetic effects than by ion saturation. All experiments were conducted in a 2 m stainless-steel coil (0.1 in inner diameter) housed in a temperature-controlled oven. A downstream backpressure regulator maintained a constant system pressure, ensuring stable operation throughout the tests. A schematic of the DSL setup is shown in Fig. 2.

The system was operated at 350 psi backpressure to prevent flashing at high temperatures and maintain consistent hydrodynamic conditions. Each test was executed in five stages:

1. A prescale run using only SW and FW to establish a baseline for the untreated system.
2. System cleaning with chemical flushing.
3. The inhibitor test phase, in which the brine mixture was dosed with a specific concentration of inhibitor.
4. Post-test cleaning to restore the system baseline
5. System reset before the next experiment.

Inhibitors were introduced by preparing the brine mixtures at the desired concentrations prior to injection. SW and FW were delivered using separate pumps and mixed at the upstream of the heated coil, where scaling reactions occurred under controlled temperature and pressure. Total flow rates were maintained at the specified values throughout each test.

Differential pressure (ΔP) across the coil was continuously monitored. Under clean conditions, ΔP remained stable, while an increase indicated deposition within the flow path. A threshold of 1 psi was used to identify the onset of deposition, and 10 psi representing significant flow restriction. The 10 psi threshold does not indicate complete blockage but serves as a reproducible basis for comparison. Accordingly, ΔP is used as an operational proxy for scaling.

All experiments were conducted with a maximum duration of 100 min. Tests reaching 10 psi were recorded as the time to scale formation, while those not reaching this threshold were reported as “ ≥ 100 min (no 10 psi reached)”. The time to reach 10 psi was used as the primary performance metric and compared with the corresponding prescale condition to evaluate inhibitor effectiveness.

### Scale characterization

To verify the nature of deposited solids responsible for pressure buildup, samples were collected from prescale tests. The deposits were analyzed using scanning electron microscopy (SEM) to examine morphology, coupled with energy-dispersive X-ray spectroscopy (EDX) to determine elemental composition. Characterization focused on prescale samples to establish the baseline scale mineralogy (e.g., sulfate scales and halite) formed from brine mixing.

### Performance evaluation criteria

Variations and increases in differential pressure may indicate a blockage in the coil. Accordingly, the effectiveness of each scale inhibitor in the DSL tests was evaluated using two key criteria: the time required to reach the set pressure differential thresholds and the minimum effective dosage needed to prevent or delay blockage.

#### Pressure differential thresholds

The selection of pressure differential thresholds in DSL testing varies across studies, with 1 psi and 10 psi being the most used benchmarks. A 1 psi increase is typically considered a conservative indicator of early-stage nucleation or potential inhibitor incompatibility, whereas 10 psi is widely used to represent advanced scale formation and significant flow restriction^21–24^.

**Fig. 3:**
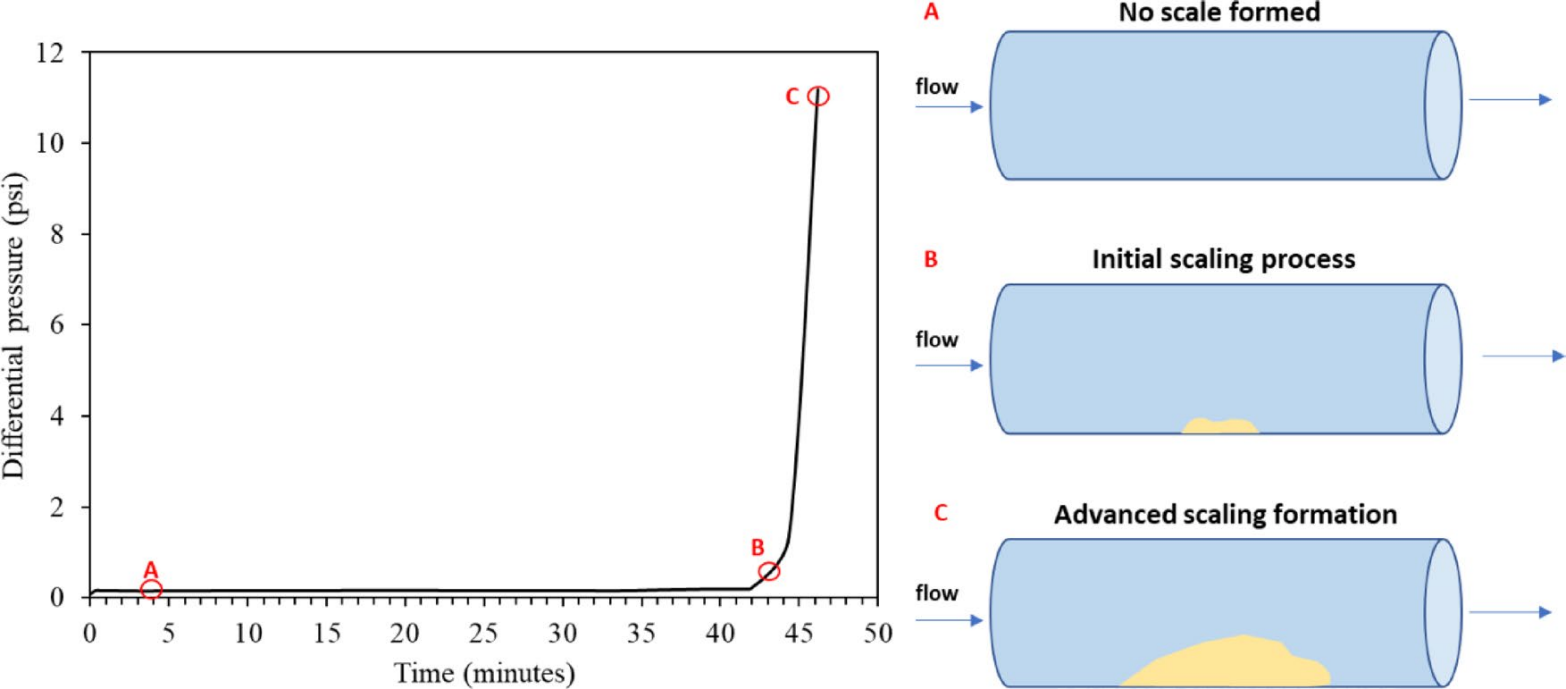
Typical DSL test profile for each stage from blank to scale formation.

In this study, 10 psi was adopted as the primary endpoint for inhibitor failure, particularly suited to high-temperature, high-salinity systems where scaling kinetics are rapid. The 1 psi threshold was additionally monitored to capture early deposition or incompatibility effects^25,26^. A typical DSL pressure profile is illustrated in Fig. 3, showing stable flow without scale (A), initial nucleation at 1 psi (B), and advanced crystal growth leading to partial tube blockage at 10 psi (C).

To enable direct comparison across different operating conditions, inhibitor performance was also expressed using a normalized improvement factor, defined as:

3 $$\text{Improvement Factor}=\frac{{t}_{\mathrm{inhibited}}}{{t}_{\mathrm{prescale}}}$$

where ${t}_{\mathrm{inhibited}}$ is the time required to reach the pressure threshold in the presence of an inhibitor, and ${t}_{\mathrm{prescale}}$ is the corresponding time under uninhibited conditions.

#### Minimum and optimum concentration

The Minimum Inhibitor Concentration (MIC) is defined as the lowest concentration at which a scale inhibitor effectively prevents the onset of scale formation under given conditions, while the Failed Inhibitor Concentration (FIC) refers to the concentration at which the inhibitor no longer provides effective protection against scaling^27–29^. Determining MIC and FIC requires establishing a time-based threshold that serves as a benchmark for performance evaluation. A commonly used reference point is the prescale time, which reflects how long it takes for scale to form in the absence of a scale inhibitor.

Although no universally accepted industry standard exists for this time boundary, some studies adopt a conservative approach, defining effective inhibition as the ability to delay scaling by at least three times the prescale time^29^. More commonly, however, inhibition is considered sufficient if the scale formation is delayed beyond the prescale duration, providing a more practical and field-aligned benchmark^3,19,25,26^. In this research, the latter criterion was selected due to its operational relevance and practicality.

## Results

This section presents the results in the following order: reproducibility, prescale (uninhibited) behavior, scale characterization, and inhibitor performance under varying conditions.

### Reproducibility of measurements

Each experimental condition was conducted in duplicate to assess reproducibility. The deviation between duplicate runs was calculated as the relative difference in the measured times to reach the pressure thresholds. Representative results for 1.0 wt% inhibitors across different temperatures and flow rates are shown in Table 2. Across all tested conditions, deviations remained below 10%. For the 1 psi threshold, deviations ranged from approximately 0.4–9.5%, with an average deviation of ~ 4.5%. Similarly, for the 10 psi threshold, deviations ranged from approximately 0.2–9.4%, with an average of ~ 4.3%.

**Table 2:** Reproducibility of DSL measurements (representative samples).

Inhibitor	Ratio	Injection rate (cc/min)	Temp (°F)	Run 1 (min)	Run 2 (min)	Deviation (%)
DTPA	50:50	1	200	57.97	59.80	3.1
GLDA	50:50	1	200	100.00	100.00	0.0
C2-SI	50:50	1	200	100.00	100.00	0.0
DTPA	50:50	10	200	5.93	6.50	9.2
GLDA	50:50	10	200	7.09	6.77	4.6
C2-SI	50:50	10	200	6.00	6.38	6.1
DTPA	50:50	1	275	60.90	64.42	5.6
GLDA	50:50	1	275	40.90	39.78	2.8
C2-SI	50:50	1	275	47.16	46.85	0.7
DTPA	50:50	10	275	5.13	5.20	1.4
GLDA	50:50	10	275	5.47	5.74	4.8
C2-SI	50:50	10	275	5.18	5.56	7.1
DTPA	50:50	1	338	81.27	87.41	7.3
GLDA	50:50	1	338	66.50	67.09	0.9
C2-SI	50:50	1	338	47.87	51.81	7.9
DTPA	50:50	10	338	5.07	5.54	8.9
GLDA	50:50	10	338	32.80	32.86	0.2
C2-SI	50:50	10	338	5.17	5.15	0.4

### Prescale test

**Fig. 4:**
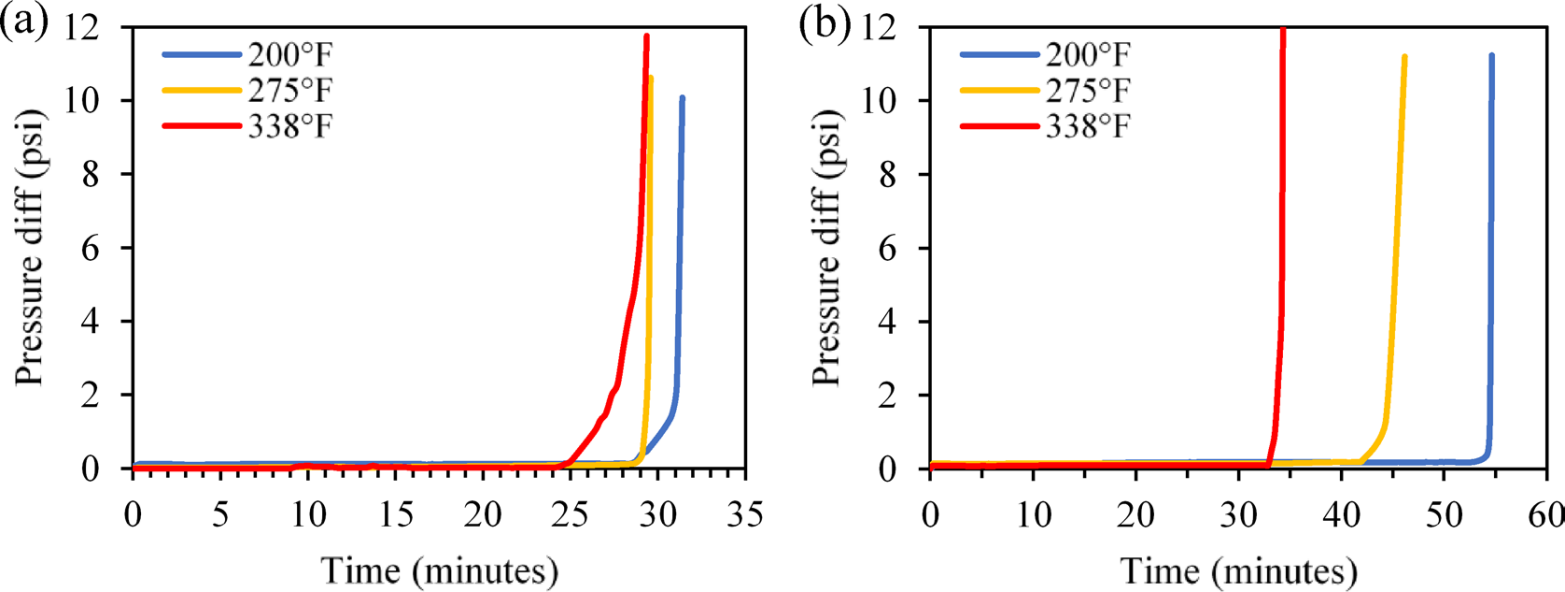
Pressure differential profiles at various temperatures under uninhibited conditions for (**a**) 50:50 and (**b**) 80:20 mixture, with a flow rate of 1 cc/min.

**Fig. 5:**
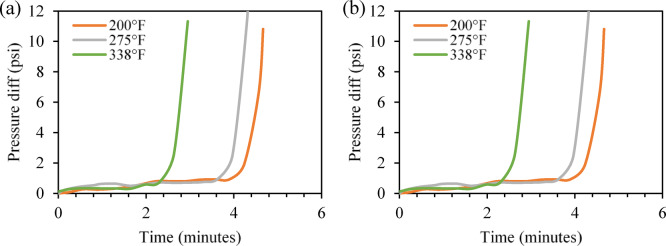
Pressure differential profiles at various temperatures under uninhibited conditions for (**a**) 50:50 and (**b**) 80:20 mixture, with a flow rate of 10 cc/min.

Figures 4 and 5 present the prescale test results for 50:50 and 80:20 mixing ratio at a total flow rate of 1 and 10 cc/min, respectively. Scale formation was strongly influenced by temperature, with higher temperatures leading to faster scale formation, indicated by a quicker time for a 10 psi pressure difference spike. For example, at an 80:20 ratio and 1 cc/min, scale formed after approximately 34 min at 338 °F, compared to 54 min at 200 °F.

Injection rate also had a significant effect. At 338 °F and a 50:50 ratio, scale formation occurred in about 3 min at 10 cc/min, compared to 29 min at 1 cc/min, indicating faster scaling at higher rates. The mixing ratio further influenced scaling behaviour. At both injection rates, the 80:20 ratio consistently delayed scale formation relative to the 50:50 ratio. For instance, at 338 °F and 10 cc/min, scale formed in approximately 5 min for the 80:20 ratio, compared to about 3 min for the 50:50 ratio.

**Fig. 6:**
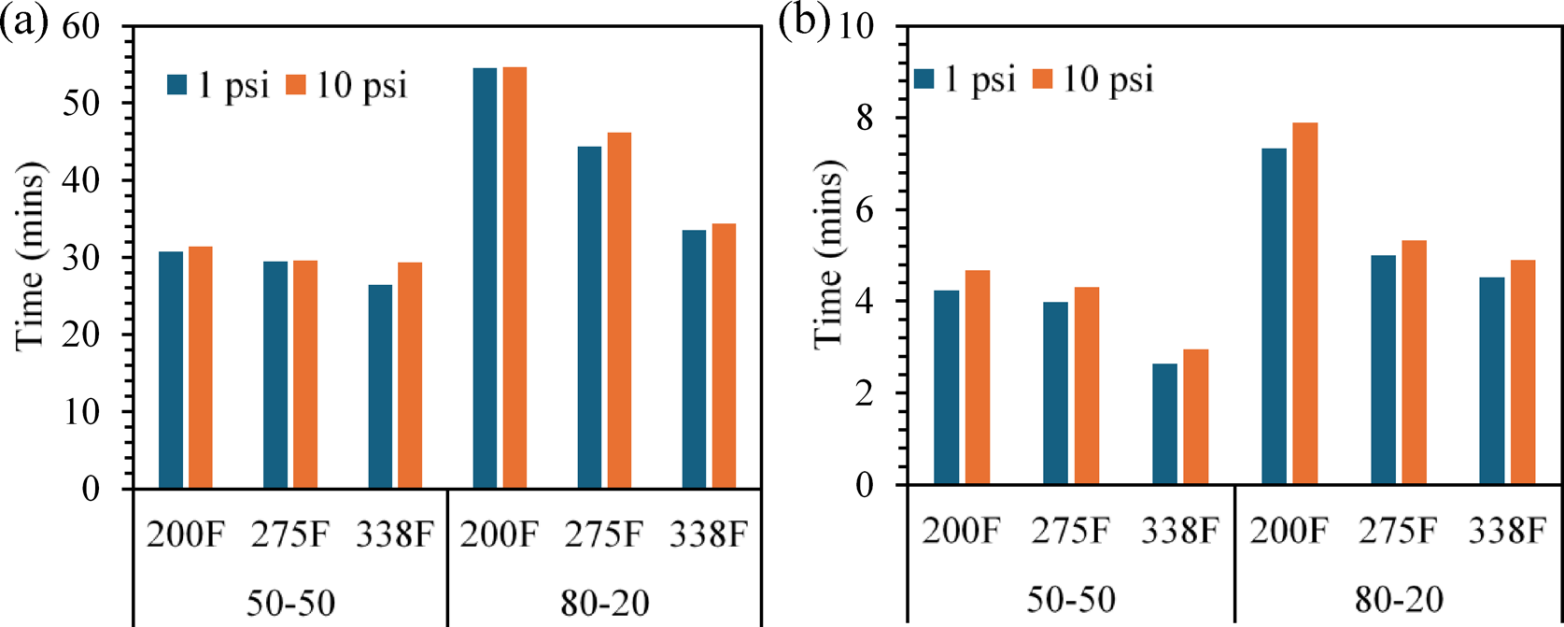
Time to reach (**a**) 1 psi and (**b**) 10 psi differential pressure under uninhibited conditions at various temperatures and flow rates. Results highlight the influence of temperature and flow on early scale nucleation (1 psi) and advanced scale formation (10 psi), with faster scaling observed at higher temperatures and a flow rate of 10 cc/min.

A pressure differential of 1 psi indicates a preliminary buildup of scale, whereas significant scale accumulation is denoted by a pressure differential of 10 psi. Figure 6 illustrates the durationfor each configuration to attain these pressure differentials.

### Scale characterization

**Fig. 7:**
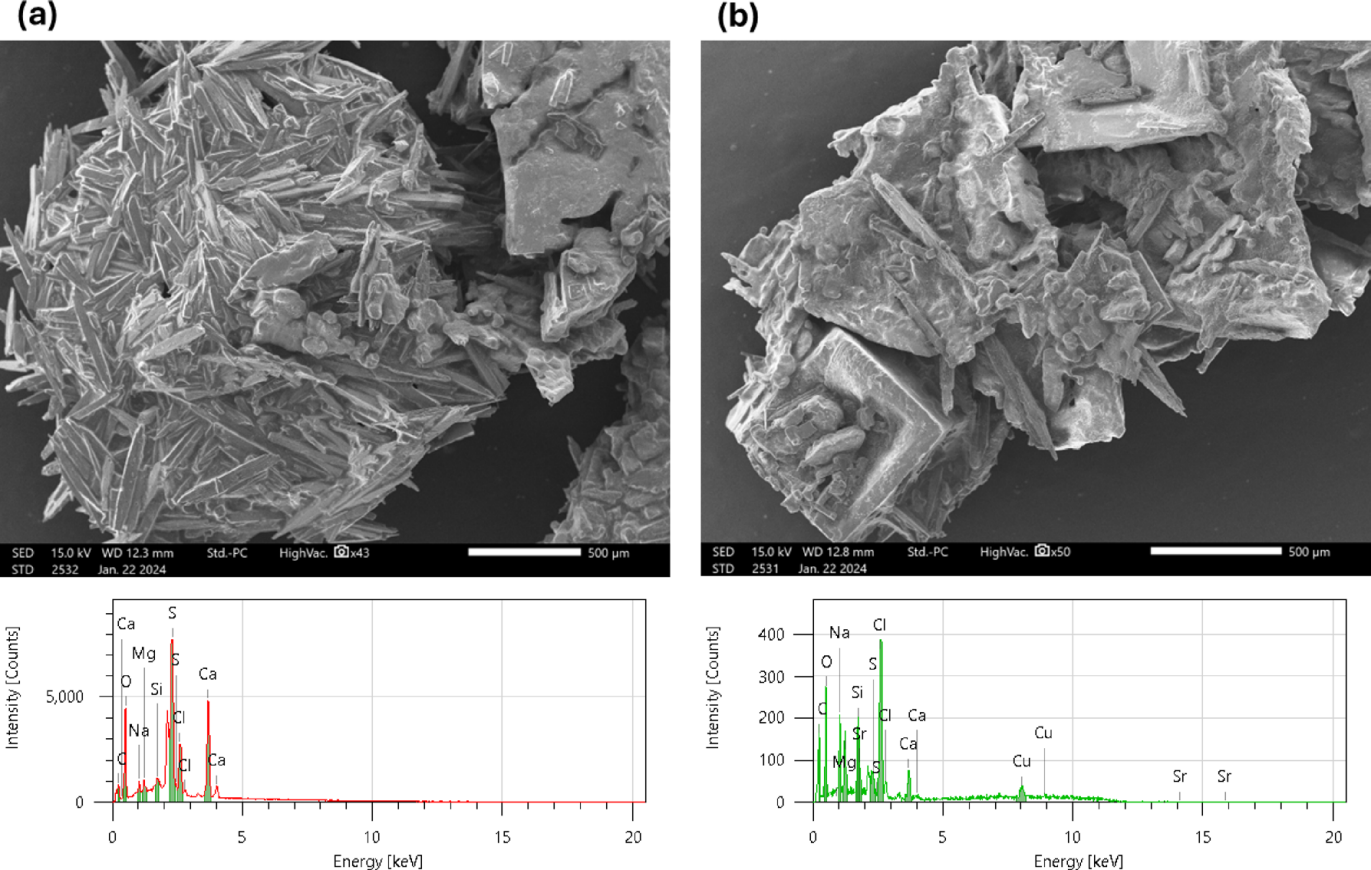
SEM images and corresponding EDX spectra of deposits from prescale tests at (**a**) 338 °F and (**b**) 200 °F.

Figure 7 shows the SEM–EDX results of deposits obtained from prescale conditions. The SEM images reveal dense crystalline structures with both elongated and plate-like morphologies. EDX spectra indicate the presence of Ba, Sr, Ca, S, Na, and Cl, suggesting that the deposits are dominated by sulfate scales with the presence of halite, consistent with previous findings^15^. At 338 °F (Fig. 7a), the deposits appear more compact and aggregated, indicating enhanced crystal growth at elevated temperature. In contrast, the 200 °F sample (Fig. 7b) shows less dense, more dispersed structures. This suggests enhanced crystal growth rates at elevated temperatures.

### Scale inhibitor test

This section presents detailed performance profiles of each scale inhibitor under the tested conditions. Pressure profiles are analyzed across temperature, mixing ratio, injection rates, and concentrations to evaluate their effectiveness in delaying scale formation.

#### DTPA

**Fig. 8:**
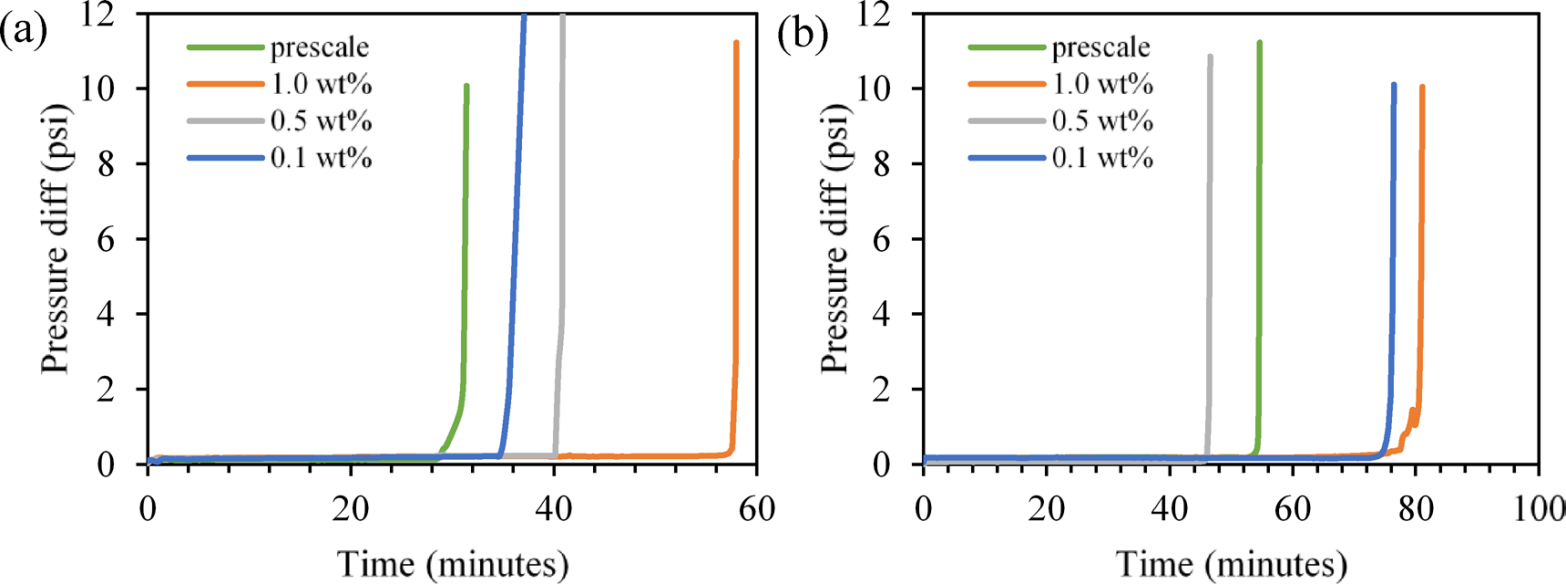
Pressure differential over time for each DTPA concentration at 200 °F and a flow rate of 1 cc/min for (**a**) 50:50 and (**b**) 80:20 mixture.

**Fig. 9:**
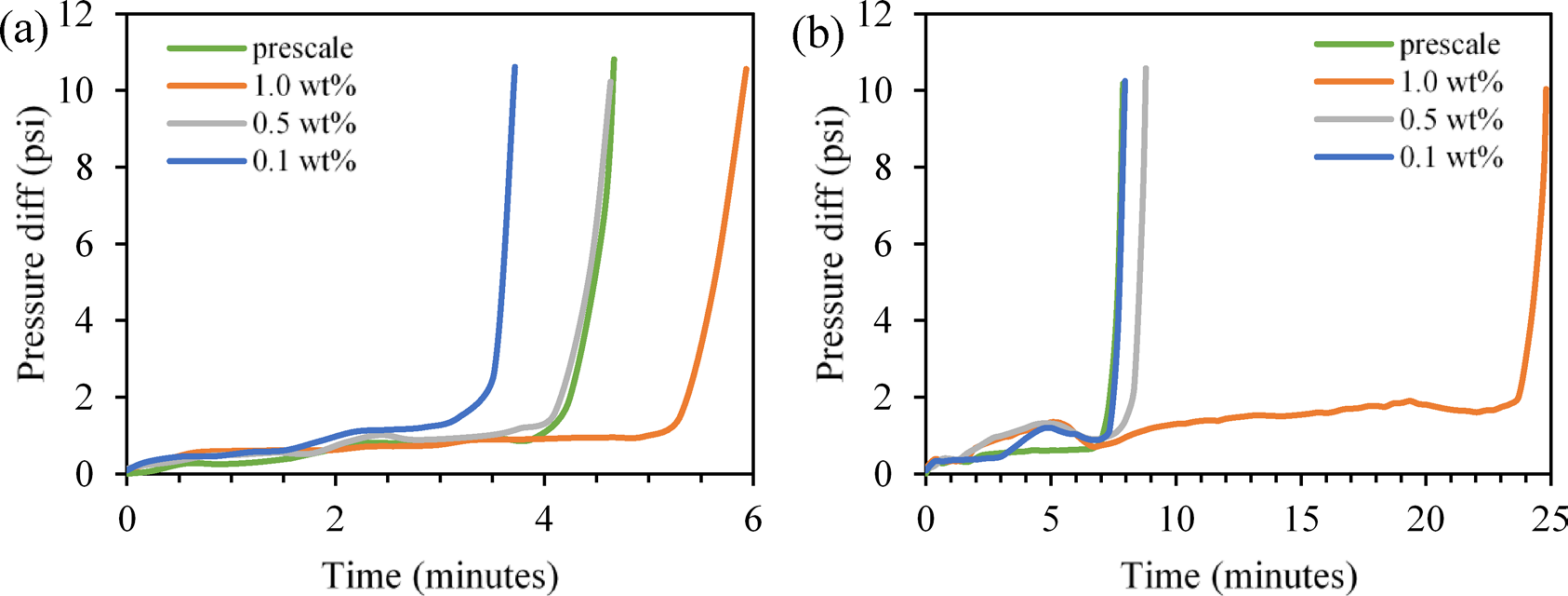
Pressure differential over time for each DTPA concentration at 200 °F and a flow rate of 10 cc/min for (**a**) 50:50 and (**b**) 80:20 mixture.

Figures 8 and 9 present the DTPA result at 200 °F for a total injection rate of 1 and 10 cc/min, respectively. Increasing DTPA concentration generally improved scale inhibition, with 1.0 wt% showing the strongest performance. At 1 cc/min, all concentrations delayed scale formation except 0.5 wt% at the 80:20 ratio. At 10 cc/min, only 1.0 wt% provided significant delay, while lower concentrations showed limited effectiveness. Overall, DTPA delayed scale formation by approximately 6–81 min relative to prescale conditions.

**Fig. 10:**
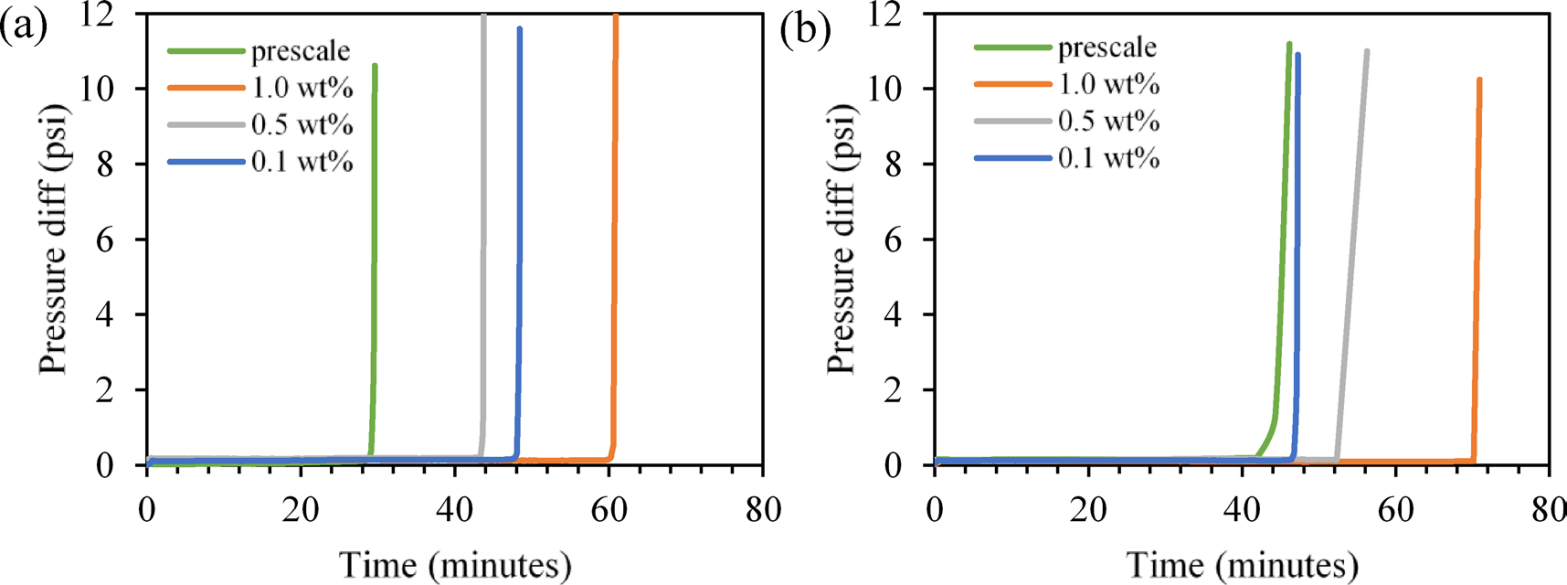
Pressure differential over time for each DTPA concentration at 275 °F and a flow rate of 1 cc/min for (**a**) 50:50 and (**b**) 80:20 mixture.

**Fig. 11:**
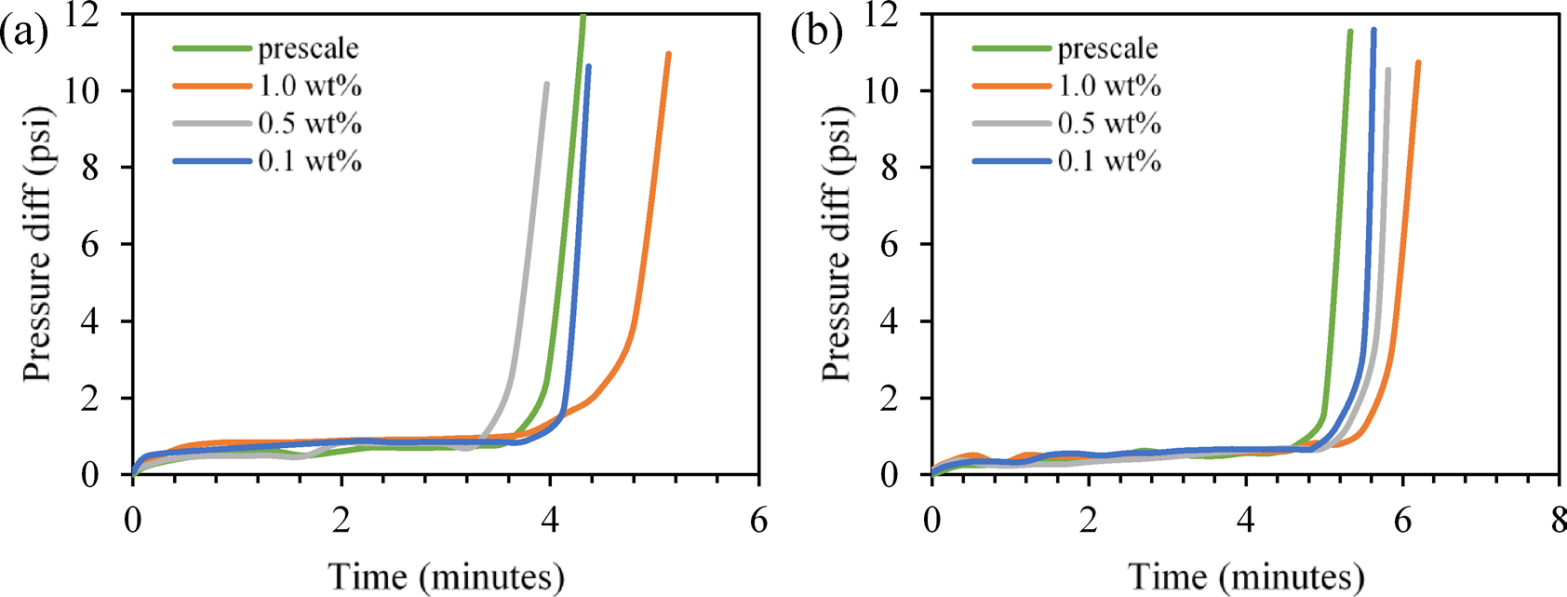
Pressure differential over time for each DTPA concentration at 275 °F and a flow rate of 10 cc/min for (**a**) 50:50 and (**b**) 80:20 mixture.

Figures 10 and 11 present the DTPA results at 275 °F for total flow rates of 1 and 10 cc/min, respectively. Similar to the behavior observed at 200 °F, increasing DTPA concentration improved inhibition performance, with 1.0 wt% providing the longest delay. At 1 cc/min, all concentrations delayed scale formation relative to prescale conditions, consistent with the trend at lower temperature but with generally shorter delays. At 1.0 wt%, scale formation occurred at approximately 61 min (50:50) and 71 min (80:20), corresponding to delays of 31 and 24 min, respectively. At 10 cc/min, the trend also followed that observed at 200 °F, where higher injection rates reduced inhibitor effectiveness. Most concentrations delayed scaling, except 0.5 wt% at the 50:50 ratio. The best performance was observed at 1.0 wt%, where scale formed at approximately 5–6 min, representing only a modest delay (~ 0.8 min) relative to prescale.

**Fig. 12:**
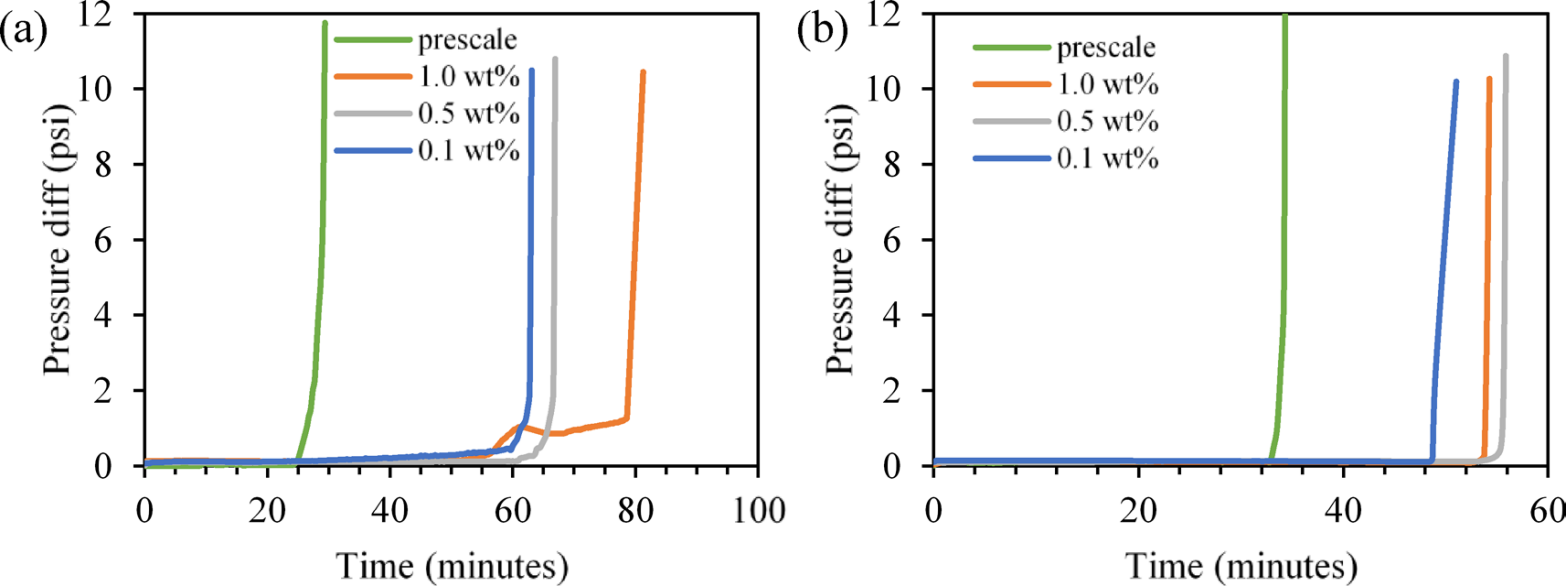
Pressure differential over time for each DTPA concentration at 338 °F and a flow rate of 1 cc/min for (**a**) 50:50 and (**b**) 80:20 mixture.

**Fig. 13:**
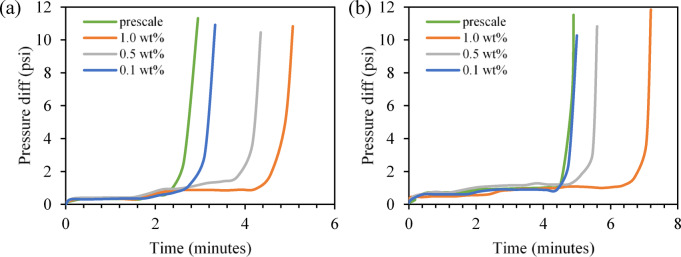
Pressure differential over time for each DTPA concentration at 338 °F and a flow rate of 10 cc/min for (**a**) 50:50 and (**b**) 80:20 mixture.

Figures 12 and 13 present the DTPA results at 338 °F for total flow rates of 1 and 10 cc/min, respectively. Consistent with the trends observed at lower temperatures, DTPA maintained effective inhibition at 1 cc/min, with all concentrations delaying scale formation beyond prescale conditions. However, the temperature increase further amplified performance differences between concentrations.

At 1 cc/min, 1.0 wt% showed the strongest performance at the 50:50 ratio, delaying scale formation by up to 81 min (52 min beyond prescale). At the 80:20 ratio, however, 0.5 wt% outperformed 1.0 wt%, delaying scale formation by 56 min (21.5 min beyond prescale), indicating a shift in optimal concentration under these conditions. At 10 cc/min, similar to lower temperatures, higher injection rates reduced overall effectiveness. All concentrations provided some delay, but improvements were limited. The best performance was observed at 1.0 wt%, delaying scale formation by approximately 3 min relative to prescale (about 5 min at 50:50 and 7 min at 80:20).

#### GLDA

**Fig. 14:**
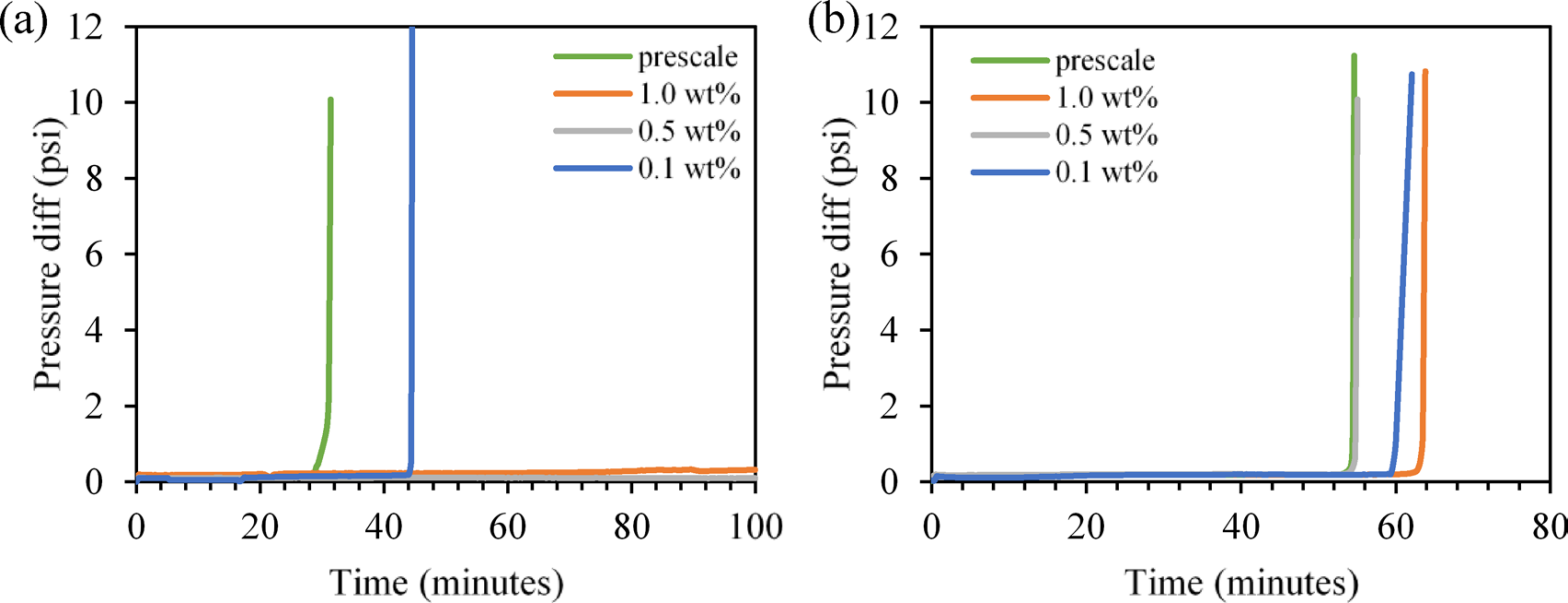
Pressure differential over time for each GLDA concentration at 200 °F and a flow rate of 1 cc/min for (**a**) 50:50 and (**b**) 80:20 mixture.

**Fig. 15:**
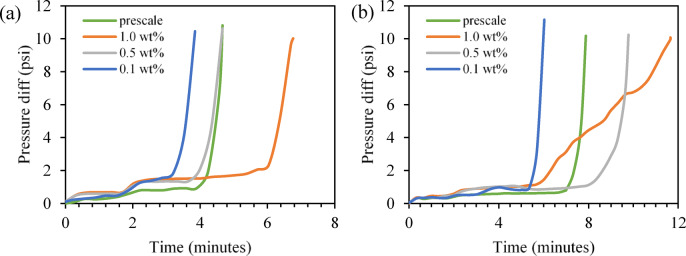
Pressure differential over time for each GLDA concentration at 200 °F and a flow rate of 10 cc/min for (**a**) 50:50 and (**b**) 80:20 mixture.

Figures 14 and 15 present the GLDA results at 200 °F for total flow rates of 1 and 10 cc/min, respectively. Increasing GLDA concentration generally improved inhibition performance, with 1.0 wt% providing the longest delay. At 1 cc/min, all concentrations delayed scale formation, with 0.5 and 1.0 wt% reaching the test limit (≥ 100 min) at the 50:50 ratio and approximately 64 min at the 80:20 ratio with 1.0 wt%. At 10 cc/min, similar to DTPA, performance was reduced, and only 1.0 wt% provided a noticeable delay, while lower concentrations showed limited improvement. In some cases, an early increase in pressure suggested possible incompatibility effects.

**Fig. 16:**
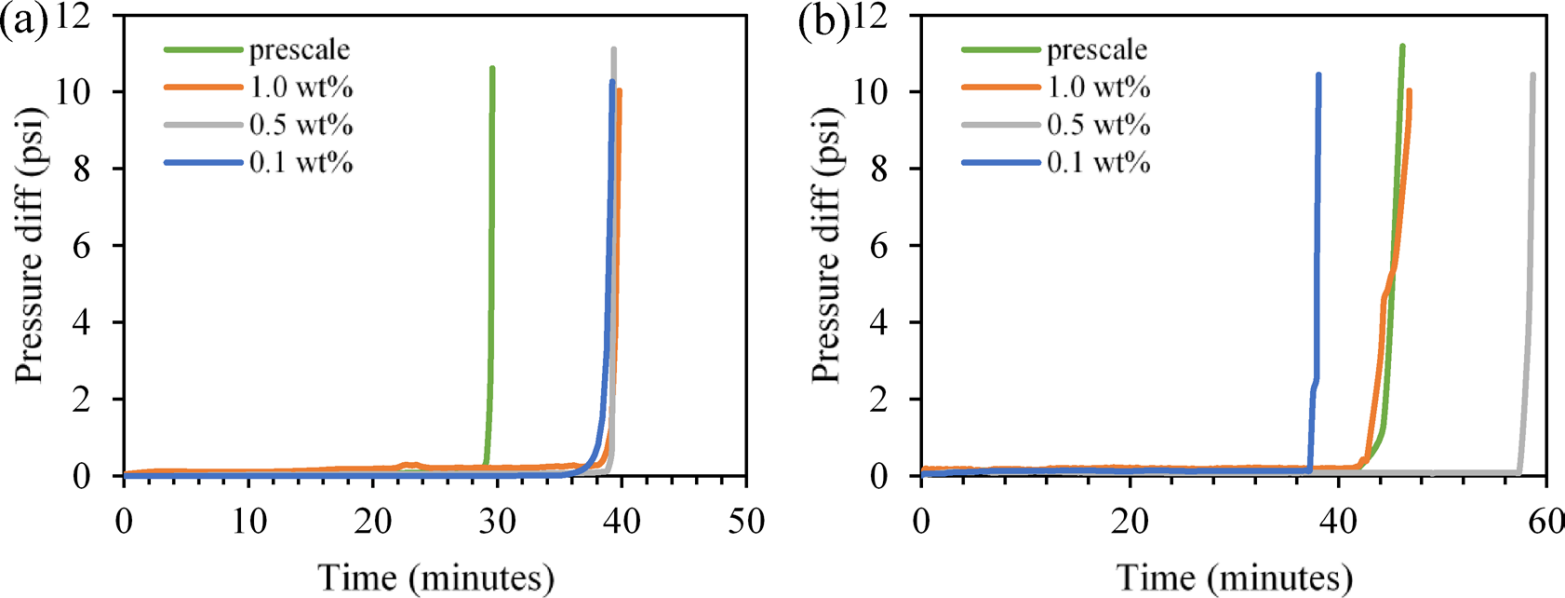
Pressure differential over time for each GLDA concentration at 275 °F and a flow rate of 1 cc/min for (**a**) 50:50 and (**b**) 80:20 mixture.

**Fig. 17:**
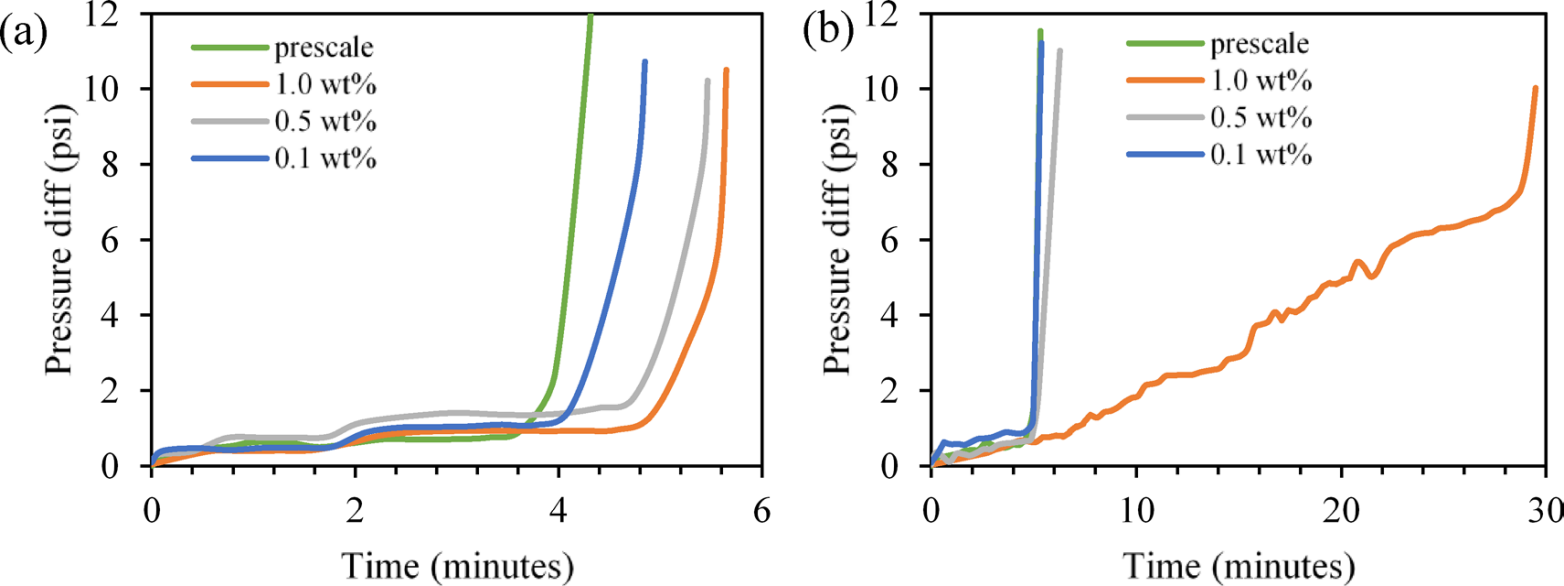
Pressure differential over time for each GLDA concentration at 275 °F and a flow rate of 10 cc/min for (**a**) 50:50 and (**b**) 80:20 mixture.

Figures 16 and 17 show the GLDA results at 275 °F for total flow rates of 1 and 10 cc/min, respectively. Compared to 200 °F, GLDA performance became more sensitive to mixing ratio and concentration. At 1 cc/min, GLDA delayed scale formation in the 50:50 ratio to ~ 39 min (9 min beyond prescale), while only 0.5 wt% was effective in the 80:20 ratio, extending the scale to 59 min (13 min longer than prescale). At 10 cc/min, all concentrations delayed scaling, with 1.0 wt% GLDA performing best. It postponed scale to 6 min in the 50:50 ratio (2 min beyond prescale) and to 29 min in the 80:20 ratio, nearly six times longer compared to prescale. However, incompatibilities were observed after 10 min, as indicated by a progressive rise in pressure differential.

**Fig. 18:**
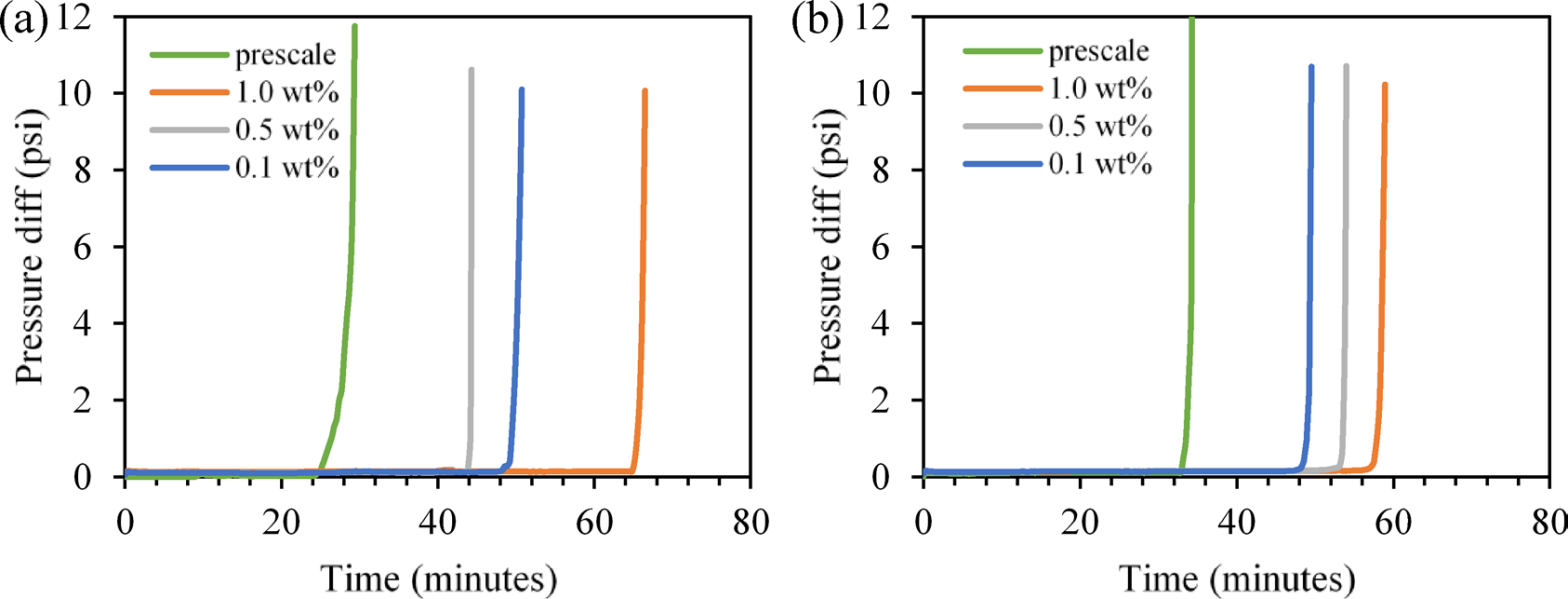
Pressure differential over time for each GLDA concentration at 338 °F and a flow rate of 1 cc/min for (**a**) 50:50 and (**b**) 80:20 mixture.

**Fig. 19:**
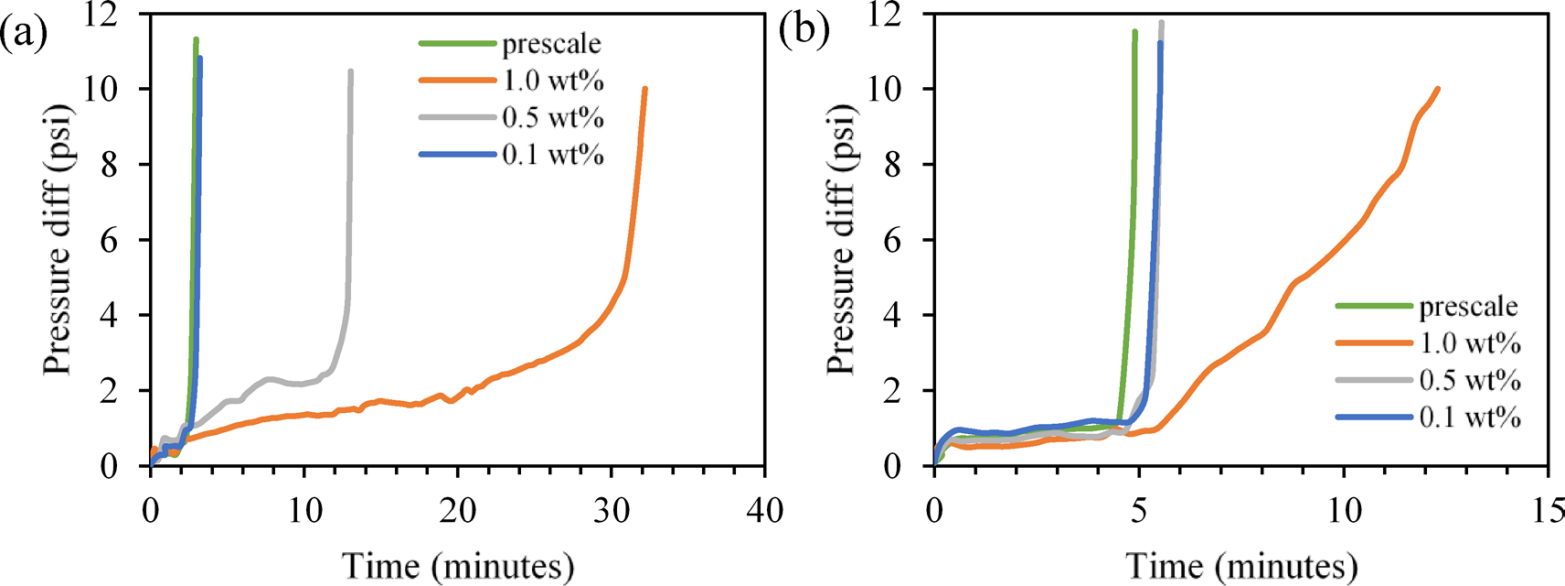
Pressure differential over time for each GLDA concentration at 338 °F and a flow rate of 10 cc/min for (**a**) 50:50 and (**b**) 80:20 mixture.

Figures 18 and 19 present the GLDA results at 338 °F for total flow rates of 1 and 10 cc/min, respectively. At this elevated temperature, GLDA maintained strong inhibition performance, particularly at higher concentrations. At 1 cc/min, all concentrations delayed scale formation beyond prescale conditions, with 1.0 wt% showing the best performance. Scale formation occurred at approximately 66 min (50:50) and 59 min (80:20).

The same behavior persisted at 10 cc/min, with 1.0 wt% GLDA demonstrating optimal performance for both ratios. The 50:50 ratio postponed scale formation to 32 min, compared to only 3 min during prescale. The 80:20 ratio resulted in a 12-min delay, compared to 5 min during prescale. Nevertheless, the initial phase, which reflected a steady, incremental increase in differential pressure, revealed incompatibilities.

#### Phosphonate-based scale inhibitor

**Fig. 20:**
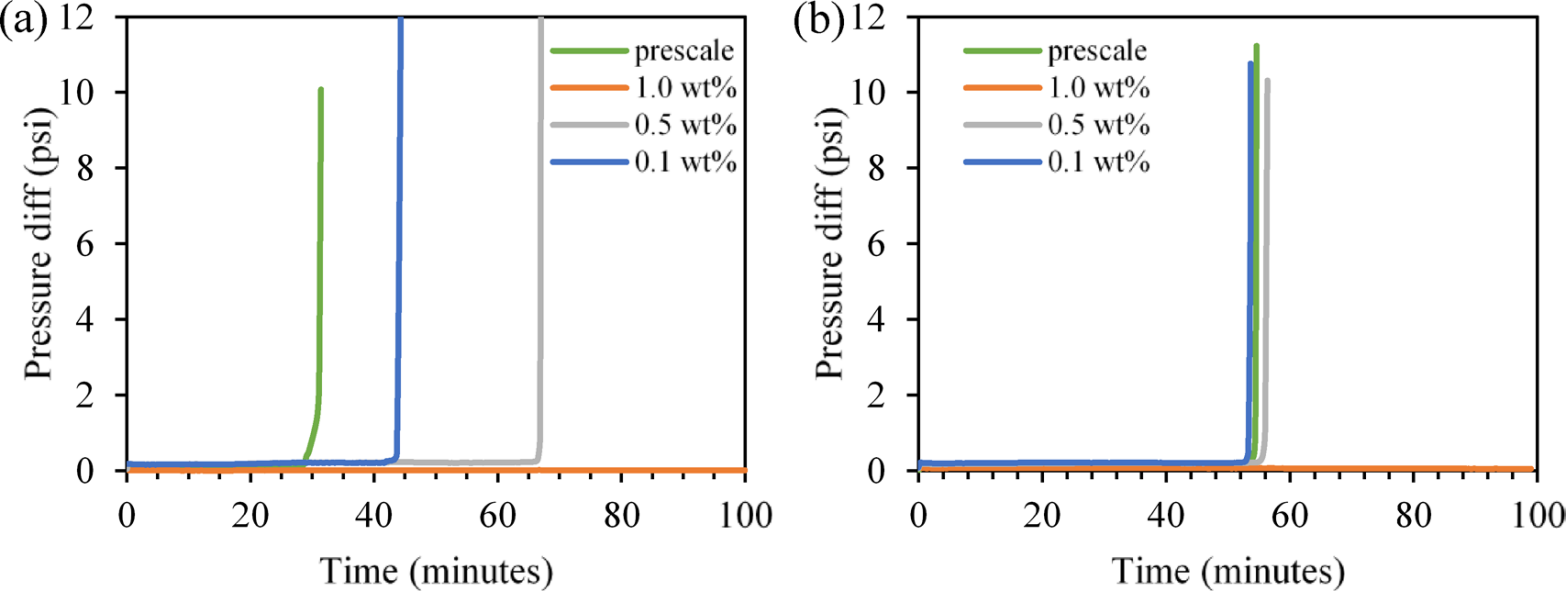
Pressure differential over time for each phosphonate SI concentration at 200 °F and a flow rate of 1 cc/min for (**a**) 50:50 and (**b**) 80:20 mixture.

**Fig. 21:**
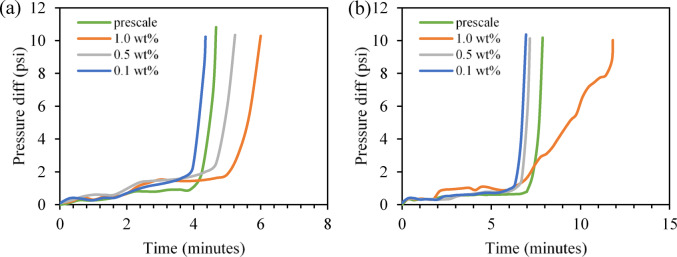
Pressure differential over time for each phosphonate SI concentration at 200 °F and a flow rate of 10 cc/min for (**a**) 50:50 and (**b**) 80:20 mixture.

Figures 20 and 21 present the phosphonate inhibitor results at 200 °F for total flow rates of 1 and 10 cc/min, respectively. At a flow rate of 1 cc/min,phosphonate SI effectively inhibited scale formation, except for the 0.1 wt% concentration in the 80:20 ratio. The findings indicated that increasing concentrations were associated with prolonged delays in scale formation. At a 1.0 wt% concentration of phosphonate SI, the test limit (≥ 100 min) was reached for both mixing ratios. At 10 cc/min, performance decreased, and only higher concentrations provided noticeable delays, while 0.1 wt% showed minimal improvement over prescale. In some cases, early pressure increases suggest possible incompatibility effects.

**Fig. 22:**
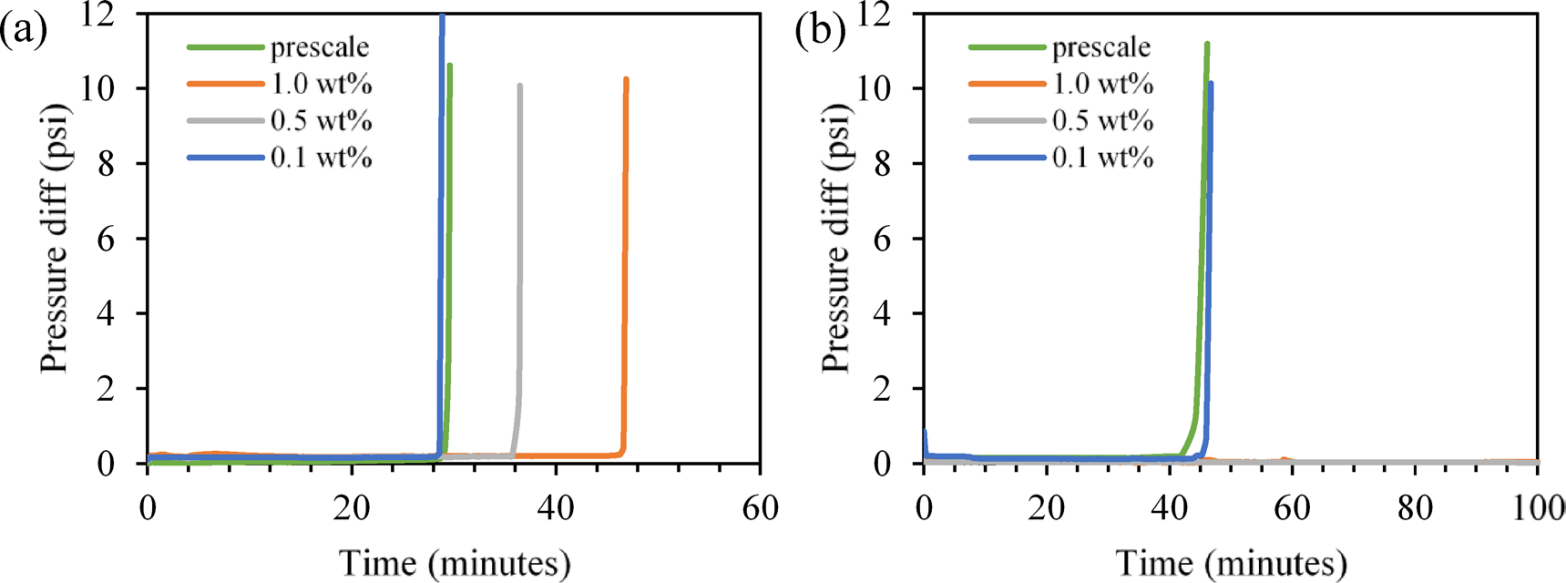
Pressure differential over time for each phosphonate SI concentration at 275 °F and a flow rate of 1 cc/min for (**a**) 50:50 and (**b**) 80:20 mixture.

**Fig. 23:**
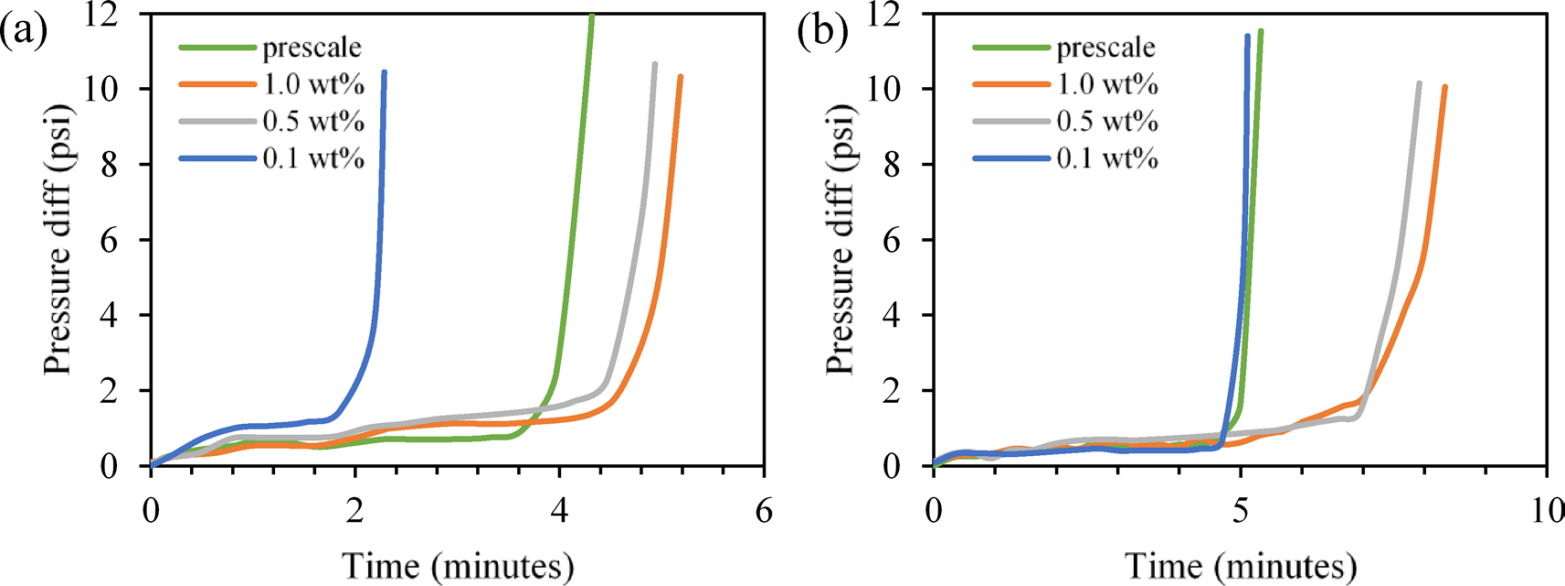
Pressure differential over time for each phosphonate SI concentration at 275 °F and a flow rate of 10 cc/min for (**a**) 50:50 and (**b**) 80:20 mixture.

Figures 22 and 23 show the phosphonate results at 275 °F for total flow rates of 1 and 10 cc/min, respectively. Similar to GLDA, performance became less consistent across conditions. At 1 cc/min, most concentrations delayed scale formation, with 0.5 wt% and 1.0 wt% reaching the test limit (≥ 100 min) at the 80:20 ratio, while performance at the 50:50 ratio was more limited. At 10 cc/min, higher concentrations continued to provide some delay, but improvements were modest, particularly at the 50:50 ratio.

**Fig. 24:**
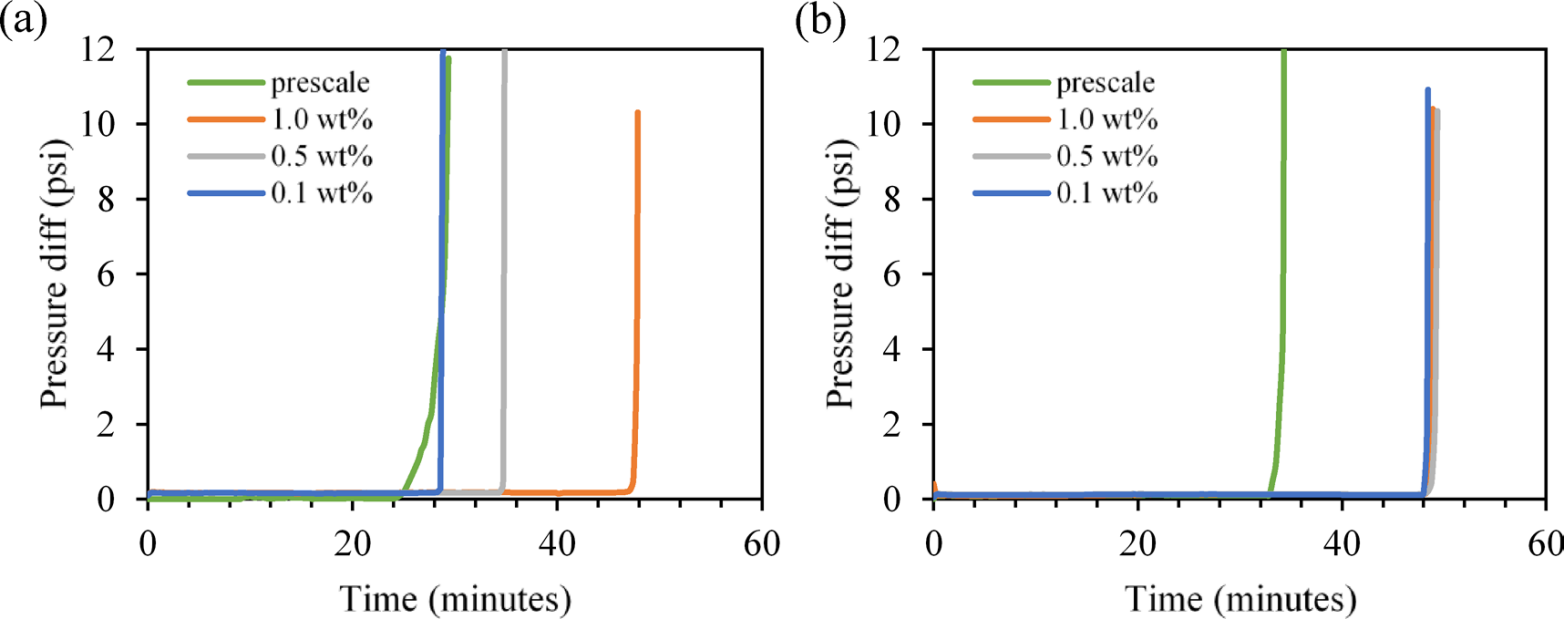
Pressure differential over time for each phosphonate SI concentration at 338 °F and a flow rate of 1 cc/min for (**a**) 50:50 and (**b**) 80:20 mixture.

**Fig. 25:**
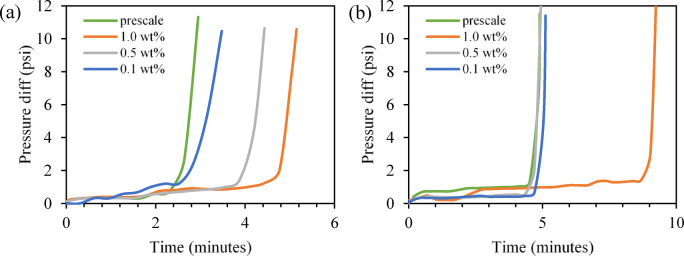
Pressure differential over time for each phosphonate SI concentration at 338 °F and a flow rate of 10 cc/min for (**a**) 50:50 and (**b**) 80:20 mixture.

Figures 24 and 25 present the phosphonate results at 338 °F for total flow rates of 1 and 10 cc/min, respectively. At this elevated temperature, the inhibitor remained effective at 1 cc/min, with all concentrations delaying scale formation beyond prescale conditions.

At the 50:50 ratio, 1.0 wt% delayed scale formation to approximately 48–52 min, while at the 80:20 ratio, performance was similar across concentrations (~ 49 min), indicating limited sensitivity to concentration under these conditions. At 10 cc/min, performance was reduced, consistent with trends observed at lower temperatures. Higher concentrations provided moderate delays, with 1.0 wt% delaying scale formation by approximately 5–9 min relative to prescale.

## Discussion and performance comparison

This section evaluates scale inhibitor performance under HTHS dynamic flow conditions. The analysis is primarily based on the time required to reach a 10 psi pressure differential, representing significant flow restriction. To complement this, the 1 psi threshold is also assessed as a sensitive indicator of initial scale nucleation or early inhibitor incompatibility.

**Fig. 26:**
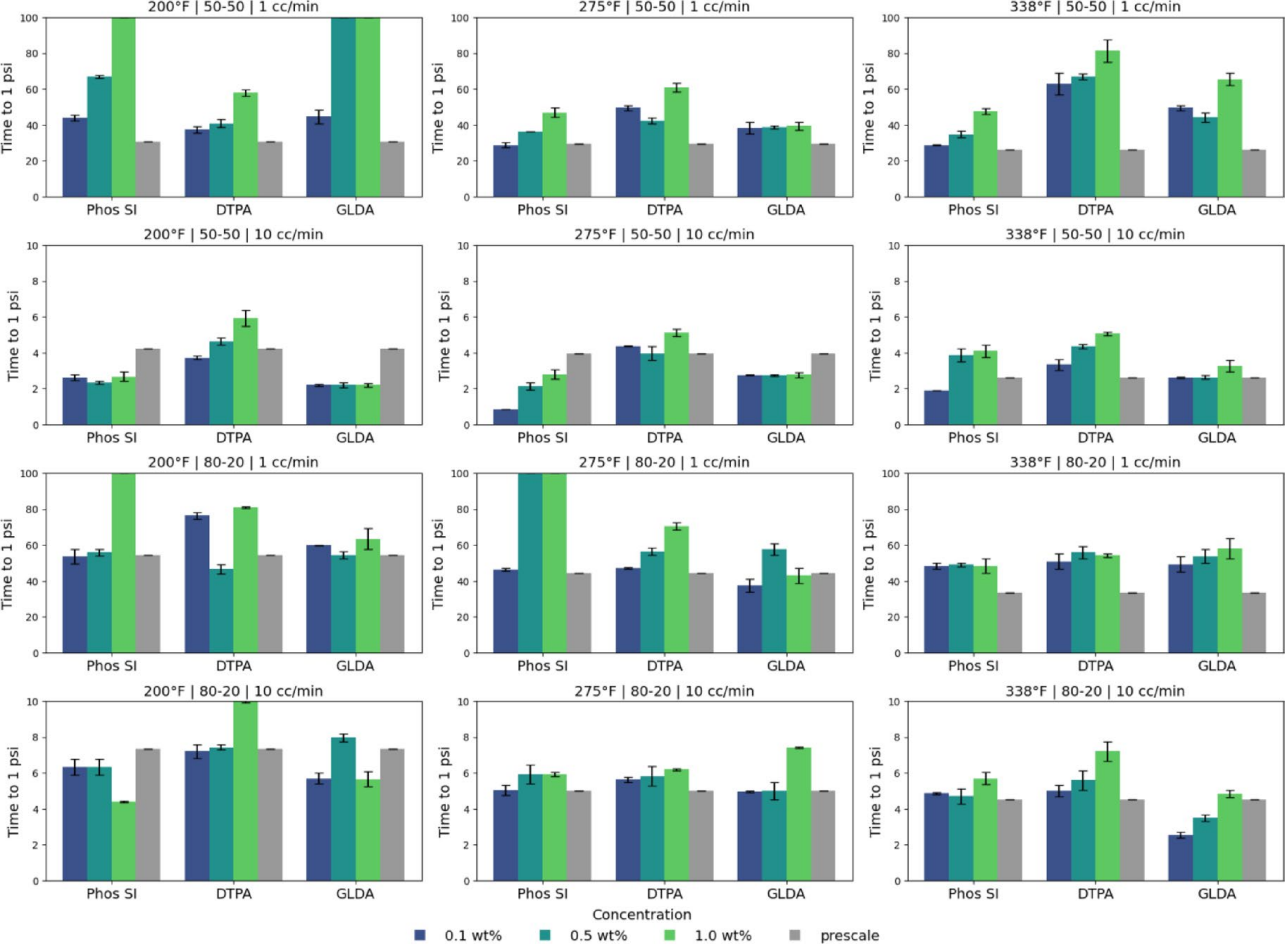
Time required to reach a 1 psi pressure differential across all tested inhibitors under varying conditions of temperature (200 °F, 275 °F, 338 °F), flow rate (1 and 10 cc/min), and mixing ratio (50:50 and 80:20). Bars represent inhibitor performance at three concentrations (0.1 wt%, 0.5 wt%, and 1.0 wt%) compared against a prescale (uninhibited) baseline. Error bars indicate variability between two replicate tests.

**Fig. 27:**
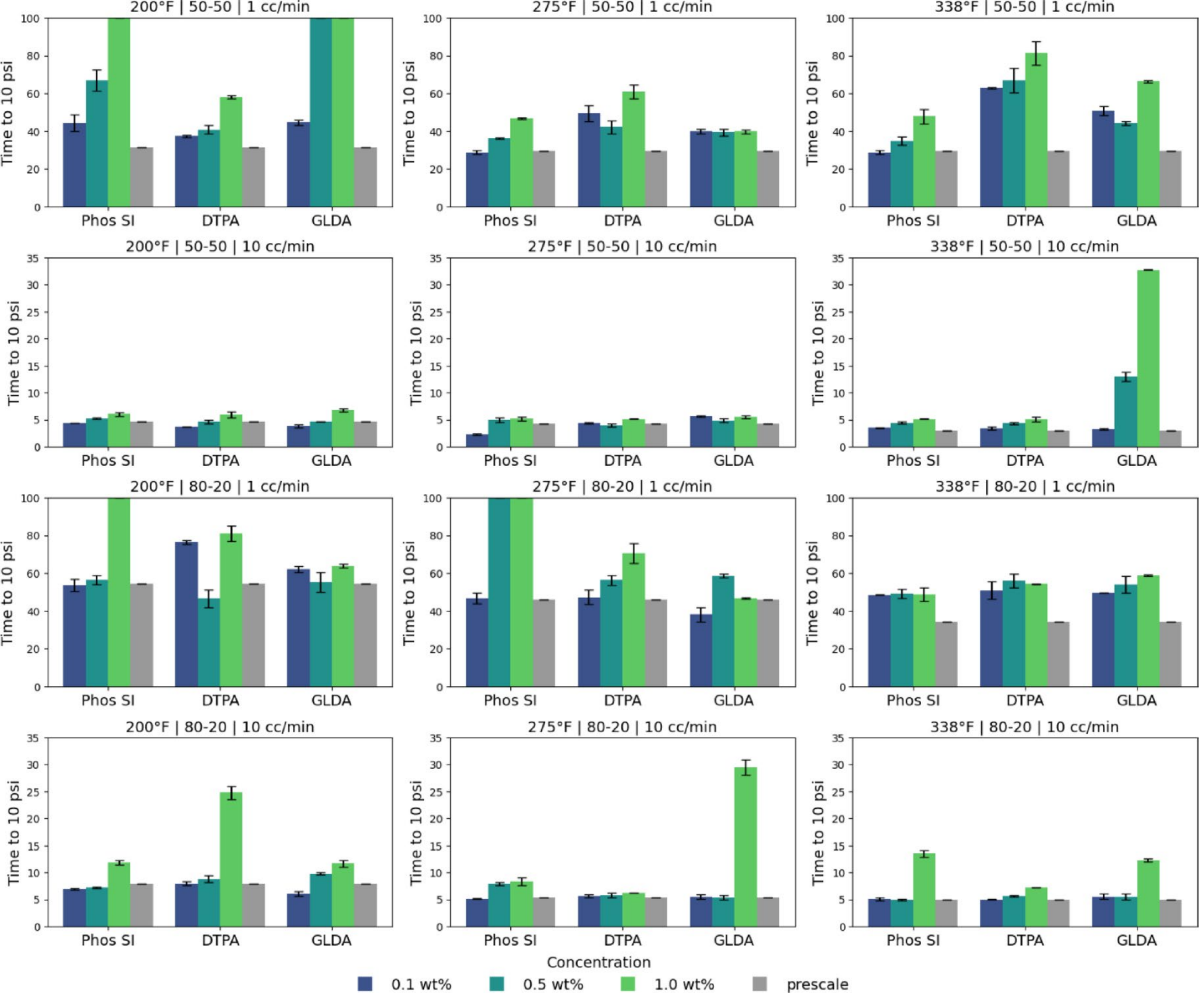
Comparative performance of DTPA, GLDA, and phosphonate scale inhibitors across various concentrations, temperatures (200 °F, 275 °F, 338 °F), mixing ratio (50:50 and 80:20), and flow rates (1 and 10 cc/min), based on the time to reach 10 psi pressure differential indicating advanced scale formation.

Overall, performance trends at 1 psi were consistent with 10 psi, indicating that early nucleation behaviour generally translates into long-term inhibition performance. However, subtle differences emerged in early-stage kinetics, particularly with phosphonate inhibitors, which in several cases delayed advanced scaling but failed to suppress initial nucleation. Figure 26 presents the comparative inhibitor performance based on the 1 psi threshold, while Fig. 27 summarizes results at 10 psi.

### Scaling behaviour under HTHS conditions

Scale formation under dynamic conditions was strongly governed by temperature, flow rate, and brine composition. Under uninhibited conditions, increasing temperature and flow rate significantly accelerated scale formation, with precipitation occurring within minutes at 338 °F and 10 cc/min. This behavior reflects enhanced ion mobility, rapid mixing, and increased supersaturation, which promote fast nucleation and crystal growth.

The effect of mixing ratio further highlights the role of ion availability. The 50:50 brine mixture produced more aggressive scaling due to increased interaction between anions and divalent cations, while the 80:20 mixture reduced scaling tendency. These baseline trends establish the severity of the scaling conditions and serve as a reference for evaluating inhibitor performance.

### Inhibition mechanism and chemical effects

The performance of the tested inhibitors reflects differences in their underlying inhibition mechanisms. Chelating agents primarily operate by reducing the availability of free divalent cations required for nucleation. In contrast, phosphonate inhibitors are generally associated with threshold inhibition and modification of crystal growth.

DTPA exhibited strong performance under high-temperature and high-supersaturation conditions, which can be attributed to its higher thermodynamic stability constants with cations. GLDA demonstrated relatively consistent performance across varying conditions, particularly at higher flow rates. This behavior suggests faster complexation kinetics, allowing GLDA to remain effective under transport-limited conditions where rapid mixing reduces the time available for inhibitor–ion interaction.

The phosphonate inhibitor showed reduced effectiveness under HTHS conditions, particularly at elevated temperatures and flow rates. This behavior is likely associated with reduced chemical stability and limited performance in high-ionic-strength environments.

### Effect of operating parameters

The influence of operating conditions on inhibitor performance reflects the interplay between thermodynamic and kinetic effects. Temperature accelerated both scaling kinetics and the demand for effective inhibition. Under these conditions, thermodynamically stable chelating agents, such as DTPA, showed improved performance compared to other inhibitors.

Injection rate introduced a transition between reaction-controlled and transport-controlled regimes. At low flow rates (1 cc/min), longer residence time allowed sufficient interaction between inhibitors and ions, enabling effective inhibition. At higher flow rates (10 cc/min), rapid mixing increased supersaturation and reduced interaction time, limiting inhibitor effectiveness.

The mixing ratio influenced scaling severity through ion availability. The 50:50 mixture created more aggressive conditions, requiring stronger inhibition mechanisms, while the 80:20 mixture allowed improved performance across most inhibitors. Inhibitor concentration generally improved performance. However, the relationship was not strictly linear, particularly under high-flow conditions where the kinetics dominated.

### Best performance inhibitor

Inhibitor performance varied substantially with temperature, mixing ratio, and injection rate.. DTPA exhibited the strongest performance under the most severe conditions, particularly at high temperature (≥ 275 °F) and 50:50 mixture, where scaling tendency was highest. Its best performance was observed at 338 °F and 1 cc/min, where 1.0 wt% DTPA delayed scale formation to over 80 min, compared to approximately 29 min under prescale conditions, representing nearly a threefold delay.

GLDA demonstrated more consistent performance across different flow rates and brine compositions. It was especially effective at higher flow rates (10 cc/min), where rapid mixing accelerates scaling in the uninhibited system. At 338 °F and 10 cc/min in the 50:50 mixture, scale formation occurred in about 3 min under prescale conditions, whereas 1.0 wt% GLDA delayed this to around 33 min, representing an order-of-magnitude improvement.

Phosphonate SI showed competitive performance under milder conditions, particularly at lower temperatures and in the 80:20 mixture at 1 cc/min, where it extended scale formation beyond the 100-min test duration compared to prescale times of ~ 50 min. However, under more severe HTHS conditions, its performance declined relative to the chelating agents, likely due to reduced thermal stability and limited effectiveness in high ionic strength environments.

To evaluate overall performance under practical conditions, the best-performing inhibitor with optimal concentration was identified for each of the 12 dynamic flow test setups. The selection was based on the longest time to reach the 10 psi pressure threshold, which serves as the primary indicator of sustained scale inhibition. Table 3 summarizes the optimal inhibitor performance across all conditions, including both the absolute time to reach 10 psi and the corresponding improvement factor relative to prescale conditions.

**Table 3:** Best-performing inhibitor and corresponding performance metrics across 12 dynamic flow setups. Each entry includes the prescale time, inhibitor performance at the 10 psi threshold and the calculated improvement (%) over the prescale.

Temperature (^o^F)	Mixing ratio (SW-FW)	Flow rate (cc/min)	Optimum concentration	Time at 10 psi (mins)	Prescale time (mins)	Improvement factor
200	50–50	1	GLDA 0.5 wt%	100.00	31.40	3.18
		10	GLDA 1.0 wt%	6.77	4.67	1.45
	80–20	1	Phos SI 1.0 wt%	100.00	54.63	1.83
		10	DTPA 1.0 wt%	24.80	7.88	3.15
275	50–50	1	DTPA 1.0 wt%	60.90	29.58	2.06
		10	GLDA 1.0 wt%	5.47	4.30	1.27
	80–20	1	Phos SI 1.0 wt%	100.00	46.17	2.17
		10	GLDA 1.0 wt%	29.48	5.33	5.53
338	50–50	1	DTPA 1.0 wt%	81.27	29.37	2.77
		10	GLDA 1.0 wt%	32.80	2.95	11.12
	80–20	1	GLDA 1.0 wt%	58.95	34.35	1.72
		10	Phos SI 1.0 wt%	13.52	4.90	2.76

All selected inhibitor formulations successfully surpassed their respective prescale times and are therefore classified as MIC under the criteria outlined in “Experimental conditions” section. This consistency underscores their effective suppression of scale formation across a diverse range of conditions. Notably, the magnitude of improvement varied substantially. Overall, the results were dominated by the chelating agents, particularly GLDA and DTPA, which emerged as the top performers in most test scenarios. In the three setups where the phosphonate-based scale inhibitor (SI) outperformed others, its performance was closely matched by one or both chelating agents, suggesting only marginal differences in those specific conditions. The most striking result was observed at 338 °F, with a 50:50 mixing ratio and 10 cc/min injection rate, where GLDA extended the time to scale formation to 32.8 min, marking an 11.12-fold improvement over the prescale baseline.

## Conclusions

The performance of chelating agents (DTPA and GLDA) as scale inhibitors under dynamic high-temperature, high-salinity conditions was systematically evaluated. The key findings are summarized as follows:

1. Scale formation under dynamic conditions was strongly influenced by temperature, flow rate, and brine composition, with more severe conditions leading to rapid scaling.
2. DTPA maintained strong performance at elevated temperatures and high-supersaturation conditions.
3. GLDA exhibited relatively consistent performance across flow rates and brine compositions, particularly under high-flow conditions where kinetic effects dominate.
4. The phosphonate inhibitor performed well under moderate conditions but showed reduced effectiveness under more severe HTHS environments.
5. Inhibitor performance is governed by both thermodynamic stability and kinetic adaptability.
6. DTPA is more suitable for high-temperature systems, while GLDA is better suited for high-flow or dynamically mixed environments.

## Data Availability

The datasets used and/or analysed during the current study are available from the corresponding author on reasonable request.

## Abbreviations

SW: Seawater
FW: Formation water
HTHS: High-temperature and high-salinity
SI: Scale inhibitor
DSL: Dynamic scale loop
TDS: Total dissolved solids
GLDA: Glutamic acid diacetic acid
DTPA: Diethylene triamine penta acetic acid
Phos SI: Phosphonate-based scale inhibitor
wt%: Weight percent

## References

[1] Olajire, A. A. A review of oilfield scale management technology for oil and gas production. *J. Pet. Sci. Eng.* **135**, 723–737 (2015).

[2] Kamal, M. S., Mohammed, M., Mahmoud, M. & Elkatatny, S. Development of chelating agent-based polymeric gel system for hydraulic fracturing. *Energies* **11**, 1663 (2018).

[3] Mpelwa, M. & Tang, S. F. State of the art of synthetic threshold scale inhibitors for mineral scaling in the petroleum industry: a review. *Pet. Sci.* **16**, 830–849 (2019).

[4] Fan, C. et al. Scale prediction and inhibition for oil and gas production at high temperature/high pressure. *SPE J.* **17**, 379–392 (2012).

[5] Li, G., Guo, S., Zhang, J. & Liu, Y. Inhibition of scale buildup during produced-water reuse: Optimization of inhibitors and application in the field. *Desalination* **351**, 213–219 (2014).

[6] Wang, W., Kan, A. T., Zhang, F., Yan, C. & Tomson, M. B. Measurement and prediction of thermal degradation of scale inhibitors. *SPE J.* **19**, 1169–1176 (2014).

[7] Murtaza, M. et al. Ionic liquid-based formulation for inhibiting calcium sulfate scale in high-pressure high-temperature wells. In *ADIPEC* (2024). 10.2118/222368-MS.

[8] Abdelgawad, K. Z., Mahmoud, M. & Hussein, I. Stimulation of high temperature carbonate gas reservoirs using seawater and chelating agents: Reaction kinetics. *J. Nat. Gas Sci. Eng.* **55**, 595–605 (2018).

[9] Almubarak, T., Ng, J. H., Ramanathan, R. & Nasr-El-Din, H. A. Chelating agents for oilfield stimulation: Lessons learned and future outlook. *J. Pet. Sci. Eng.* **205**, 108832 (2021).

[10] Barri, A., Hassan, A. & Mahmoud, M. Carbonate stimulation using chelating agents: Improving the treatment performance by optimizing the fluid properties. *ACS Omega* **7**, 8938–8949 (2022). 35309487 10.1021/acsomega.1c07329 PMC8928500

[11] Budiman, O. & Alajmei, S. Seawater-based fracturing fluid: A review. *ACS Omega*10.1021/ACSOMEGA.3C05145 (2023). 37969974 10.1021/acsomega.3c05145 PMC10633887

[12] Barber, M. & Heath, S. A new approach to testing scale inhibitors in mild scaling brines—Are dynamic scale loop tests needed? In *Proceedings—SPE International Symposium on Oilfield Chemistry* Vol. 2019 (2019).

[13] Murtaza, M. et al. Single step calcium sulfate scale removal at high temperature using tetrapotassium ethylenediaminetetraacetate with potassium carbonate. *Sci. Rep.* **12**, 1–17 (2022). 35710805 10.1038/s41598-022-14385-6 PMC9203783

[14] Ruan, G., Liu, Y., Kan, A. T., Tomson, M. B. & Zhang, P. Sodium chloride (halite) mineral scale threat assessment and scale inhibitor evaluation by two common jar test based methods. *J. Water Process Eng.* **43**, 102241 (2021).

[15] Budiman, O. et al. Scale inhibition efficiency of chelating agents under high temperature and salinity. *Arab. J. Sci. Eng.* **2025**, 1–25. 10.1007/S13369-025-10640-W (2025).

[16] Oliveira, D. F. et al. Characterization of scale deposition in oil pipelines through X-Ray microfluorescence and X-Ray microtomography. *Appl. Radiat. Isot.* **151**, 247–255 (2019). 31228733 10.1016/j.apradiso.2019.06.019

[17] Webster, N. A. S., Gan, B. K. & Livk, I. In situ X-ray diffraction analysis of the onset of mineral scale deposition from synthetic oilfield processing waters. *Fuel* **137**, 211–215 (2014).

[18] Mady, M. F., Malmin, H. & Kelland, M. A. Sulfonated nonpolymeric aminophosphonate scale inhibitors—improving the compatibility and biodegradability. *Energy Fuels* **33**, 6197–6204 (2019).

[19] Mady, M. F. & Kelland, M. A. Overview of the synthesis of salts of organophosphonic acids and their application to the management of oilfield scale. *Energy Fuels* **31**, 4603–4615 (2017).

[20] Anderegg, G., Arnaud-Neu, F., Delgado, R., Felcman, J. & Popov, K. Critical evaluation of stability constants of metal complexes of complexones for biomedical and environmental applications (IUPAC Technical Report). *Pure Appl. Chem.* **77**, 1445–1495 (2005).

[21] Ahmed, M. A. & Mohamed, A. A. Evaluation and optimization of antiscalant substances for enhanced reverse osmosis performance. *J. Saudi Chem. Soc.* **28**, 101923 (2024).

[22] Al Nasser, W. & Al Jeshi, Y. Inline technique to evaluate the performance of scale inhibitors for calcium carbonate scale aggregation and prevention. *Chem. Eng. Res. Des.* **162**, 39–44 (2020).

[23] Bhandari, N., Bhandari, M., Littlehales, I. & Fidoe, J. Development of a novel iron sulfide scale inhibitor for onshore US application. In *Proceedings—SPE International Symposium on Oilfield Chemistry* Vol. 2019 (2019).

[24] Mady, M. F. et al. Phosphonated lower-molecular-weight polyethyleneimines as oilfield scale inhibitors: An experimental and theoretical study. *Ind. Eng. Chem. Res.* **61**, 9586–9599 (2022).

[25] Sorbie, K. S. & Laing, N. How scale inhibitors work: Mechanisms of selected barium sulphate scale inhibitors across a wide temperature range. In *Proceedings - SPE Sixth International Symposium on Oilfield Scale; Exploring the Boundaries of Scale Control* 447–456 (2004). 10.2118/87470-MS.

[26] Mady, M. F. Oilfield scale inhibitors: Synthetic and performance aspects. In *Water-Formed Deposits: Fundamentals and Mitigation Strategies* 325–352 (2022). 10.1016/B978-0-12-822896-8.00033-9.

[27] Mady, M. F., Charoensumran, P., Ajiro, H. & Kelland, M. A. Synthesis and characterization of modified aliphatic polycarbonates as environmentally friendly oilfield scale inhibitors. *Energy Fuels* **32**, 6746–6755 (2018).

[28] Vazquez, O. *Modelling Oilfield Scale Squeeze Treatments: from Core to Reservoir* (Springer, 2023).

[29] Wang, Q. et al. Laboratory study on efficiency of three calcium carbonate scale inhibitors in the presence of EOR chemicals. *Petroleum* **4**, 375–384 (2018).

